# *Actaea racemosa* L. Is More Effective in Combination with *Rhodiola rosea* L. for Relief of Menopausal Symptoms: A Randomized, Double-Blind, Placebo-Controlled Study

**DOI:** 10.3390/ph13050102

**Published:** 2020-05-21

**Authors:** Lali Pkhaladze, Nina Davidova, Archil Khomasuridze, Ramaz Shengelia, Alexander G. Panossian

**Affiliations:** 1 I.Zhordania Institute of Reproductology, 13, Tevdore Mghvdeli street, 0112 Tbilisi, Georgia; davidovanino@gmail.com (N.D.); archilk@list.ru (A.K.); 2 Department for History of Medicine and Bioethics, Tbilisi State Medical University, Vazha-Pshavela avenue 33, 0162 Tbilisi, Georgia; r.shengelia@tsmu.edu; 3 Phytomed AB, Bofinkvagen 1, 31275 Vaxtorp, Sweden

**Keywords:** black cohosh, rhodiola, menopause, clinical trial

## Abstract

*Background*: The aim of this study was to assess the efficacy and safety of a new herbal preparation (Menopause Relief EP^®^), the hybrid combination of *Actaea racemosa* L. (black cohosh, BC) and *Rhodiola rosea* L. (RR) root extracts, compared with the most effective dose of BC extract in women with menopausal complaints. *Methods:* A total of 220 women were randomly assigned to receive two capsules either BC (6.5 mg), BC500 (500 mg), Menopause Relief EP^®^ (206,5), or placebo once per day for 12 weeks. The efficacy endpoints were relief of menopausal symptoms, measured using the Kupperman Menopausal Index (KMI), Menopause Relief Score (MRS), and menopause Utian Quality of Life (UQOL) index. *Results:* The menopause symptom relief effects of RR-BC were significantly superior in all tests to the effects of BC and placebo after their repeated administration for 6 and 12 weeks. There was no statistically significant difference between the effects of BC and BC500 over time. RR-BC significantly improved the QOL index in patients, compared to BC, BC500, and placebo, mainly due to the beneficial effects on the emotional and health domains. *Conclusions:* BC is more effective in combination with RR in relief of menopausal symptoms, particularly psychological symptoms.

## 1. Introduction

Menopause is а normal transition state from the reproductive into the non-reproductive phase in women, presumably associated with a decline in the production of sex hormones [1]. In four out of five women, menopause is accompanied by а wide range of vasomotor (e.g., hot flushes, sweating), psychological/vegetative (e.g., insomnia, nervousness/irritability, depressive events, and palpitation), somatic (e.g., joint pain), and urogenital/sexual (e.g., libido changes, dyspareunia, and vaginal dryness) symptoms [2]. Long-term hormonal replacement therapy has been shown to be associated with an increased risk of developing breast cancer [3]. In this context, there is a high demand for relatively safe herbal preparations that can relieve or prevent menopausal symptoms, as more than 1.2 billion women globally will experience menopause syndrome by the year 2030 [4].

Black cohosh (BC), *Actaea racemosa* L. (syn. *Cimicifuga racemosa* (L.) Nutt.), a plant native to the eastern United States, was used by North American indigenous people for gynecological conditions such as pain associated with premenstrual syndrome (PMS), pain during childbirth, and menopausal complaints, including migraines [5]. Black cohosh has traditionally been used in China, Europe, and North America for centuries to treat a variety of illnesses, including menopausal symptoms and osteoporosis [6]. Since the early 20th century, medical practitioners in the United States and Europe have used a tincture derived by macerating fresh black cohosh rhizome in a 50% alcohol solution for 10 days at a dose corresponding to 65 mg of the crude drug to reduce the frequency and severity of hot flushes and sweating in menopausal women [5]. Several *Cimicifuga racemosa* (L.) Nutt. rhizome preparations (e.g., dry genuine extract (DER) 4.5–8.5:1, extraction solvent ethanol 60%, *v/v*) are commonly known as herbal medicinal products with well-established uses in the relief of menopausal complaints such as hot flushes and profuse sweating, at a daily dose of 4.7–8.9 mg, corresponding to 40 mg of herbal substance [7,8]. Information on their posology has been derived from long-standing use, as well as recommendations contained in the German Commission E monograph (daily dose: 40 mg herbal substance) [9] and ESCOP monograph (daily dose: 40–140 mg herbal substance) [10], and has been confirmed in many clinical studies [2,8,9,10,11,12,13,14,15]. However, the most effective dose of BC has not yet been clinically estimated, although one study [11] demonstrated superior efficacy of a daily dose (i.e., 13 mg of the extract corresponding to 80 mg herbal substance) that is higher than the formal recommendation (i.e., 40 mg herbal substance) of the European Medical Agency (EMA) for manufacturers of HMP [7,8]. Furthermore, numerous black cohosh preparations are currently available for purchase in significantly higher doses, with the assumptions that they will be more effective, e.g., Black Cohosh for Women’s Natural Change (545 mg), *Cimicifuga racemosa* root and root extract (800 mg), or Black Cohosh (1000 mg) [16,17].

Therefore, one of the two aims of our study was to compare the daily dose that was found to be the most effective in the literature (13 mg) [11,12] with a dose of 1000 mg, which was compatible with the highest dose available for purchase at the time of writing.

In addition to its effectiveness in treating menopause symptoms, BC also exhibits anti-inflammatory, antidiabetic, antiviral, antioxidant, antiangiogenic, vasodilating, and immunosuppressive effects [6]. Several preclinical studies suggest its potential use in osteoporosis and Alzheimer’s disease [6]. On the other hand, the use of BC preparations for patients with a history of treated breast cancer or hormone-dependent tumors is not recommended and should be avoided [8]. Furthermore, hepatotoxic, neurotoxic, and cardiotoxic effects of BC have also been reported [6,8]. It is not yet clear which of the more than 400 biologically active compounds of BC extracts exhibit beneficial or toxic effects [5,6], despite some of these (e.g., isoferulic acid, isoflavonoid formonometin, and the major constituents, cycloartane-type tetracyclic triterpenes) being used for standardization of BC extracts [6,18].

Moreover, adaptogenic plants are known to be neuroprotective, hepatoprotective, and cardioprotective [19,20,21]. As an example, *Rhodiola rosea* L. (RR, commonly, rose root, roseroot, Arctic root, golden root) exhibits neuroprotective, cardiovasculoprotective, antistress, and anticarcinogenic effects, which demonstrate significant value in counteracting menopausal symptoms [22]. It is highly unlikely that *R. rosea* will increase the risk of cancer in hormone-sensitive tissues [22].

Furthermore, it has been reported that RR extracts and salidroside, an active constituent of the extract, inhibit the growth of human breast cancer in vitro and in vivo [23,24,25]. In this context, the combination of BC with RR is favorable in terms of the benefit/risk assessment.

Climacteric symptoms (Table A1a in Appendix A) are formally divided into four categories (vasomotor, neuropsychological/vegetative, somatic, and urogenital/atrophy) [26,27,28,29], including three groups of neuropsychological symptoms (nervousness/irritability, depressive mood, anxiety, and impaired performance/memory). These symptoms have been successfully treated with adaptogens [30,31,32,33,34], and particularly with *Rhodiola rosea* L. [35,36,37,38] dry extract (DER 1.5−5:1, extraction solvent ethanol 70%, *v/v*), which is a traditional herbal medicinal product indicated for the temporary relief of stress, fatigue, and weakness in adults at a daily dose of 144–400 mg [39]. *R. rosea* is used in recognized officinal medicines in various countries [35,39]. Numerous animal and human studies of RR show that it can improve many neuropsychological symptoms, such as fatigue, anxiety, depression, cognitive dysfunction, memory decline, reduced executive function, and stress intolerance [35,36,37,38].

Therefore, we hypothesized that a combination of BC with RR in a new herbal preparation, BC-RR (Menopause Relief EP^®^), could be more effective then BC alone in the relief of menopausal complaints in adult climacteric females.

Consequently, the primary objective of this study was to compare for the first time the efficacy of Menopause Relief EP^®^ capsules (RR-BC), containing as an active pharmaceutical ingredient a new fixed combination of *Cimicifuga* EP-40 and *Rhodiola* EPR-7 extracts [40], with *Cimicifuga* EP-40 extract (BC) taken at a daily dose of 13 mg (BC) and in a significantly higher daily dose of 1000.0 mg (BC500), in 220 adult women with menopausal complaints in a randomized, double-blind, placebo-controlled, four-arm parallel group study.

## 2. Results

### 2.1. Study Participants, Their Disposition, and Baseline Variables

**Figure 1:**
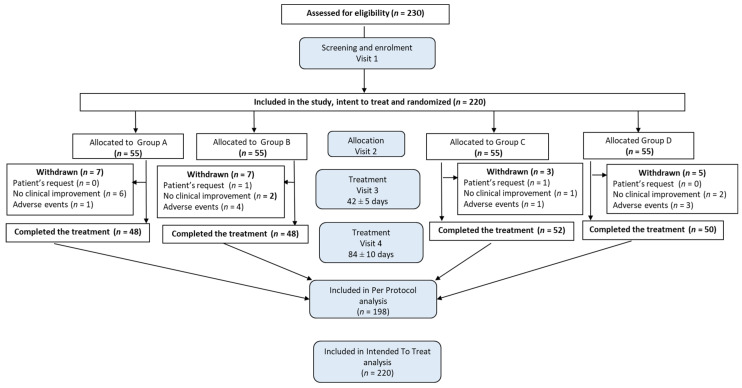
CONSORT flow chart of the disposition of patients in four arms of the study, from Visit 1 to Visit 4, followed by per protocol (PP) and intent-to-treat (ITT) analysis (Figure A1, Figure A2, Figure A3, Figure A4, Figure A5 and Figure A6 and Table A2, Table A3, Table A4, Table A5, Table A6 and Table A7). The figure shows the numbers of patients who were randomized, entered, discontinued, and completed the study as well as the reasons for all post-randomization discontinuations.

Recruitment for the study was initiated on 5 May 2018, and the last person was recruited on 24 May 2019. A total of 230 female patients were assessed for eligibility; of these, 220 (95.65%) patients with menopausal complaints met the inclusion criteria and were randomized to the RR-BC (*n* = 55), BC (*n* = 55), BC500 (*n* = 55), and placebo (*n* = 55) groups. Among the enrolled participants, 22 (10.0%) were withdrawn during the treatment phase of the study upon patient request (*n* = 2; 0.91%), or due to adverse events (*n* = 9; 4.09%) or lack of clinical improvement (*n* = 11; 5.00%). A total of 198 patients (90.00%) completed their respective treatment cycles according to the protocol. The difference in distribution of patients who completed the study between groups (RR-BC, *n* = 48; BC, *n* = 48; BC500, *n* = 52; and placebo, *n* = 50) was not statistically significant (>0.05, chi-square test). All enrolled patients were evaluated for treatment efficacy on an intent-to-treat (ITT) basis and comprised the primary efficacy subset. The disposition of patients and distribution between study groups are shown in Figure 1 and Appendix A.

Table 1a,b shows the baseline demographics and clinical characteristics of the study patients. The median age of the patients was 52.0 years at the time of enrolment. At the time of randomization, all patient characteristics were well balanced between the groups (Table 1a,b).

The groups did not show statistically significant differences in age, body mass, or Menopause Relief Scale (MRS), Kupperman Menopausal Index (KMI), or Utian Quality of Life (UQOL) index scores. We found no significant difference in baseline values for laboratory measurements, indicating successful randomization of study preparations between the groups (Table 1a,b).

### 2.2. Efficacy of Treatment

#### 2.2.1. Primary Efficacy Endpoint

**Figure 2:**
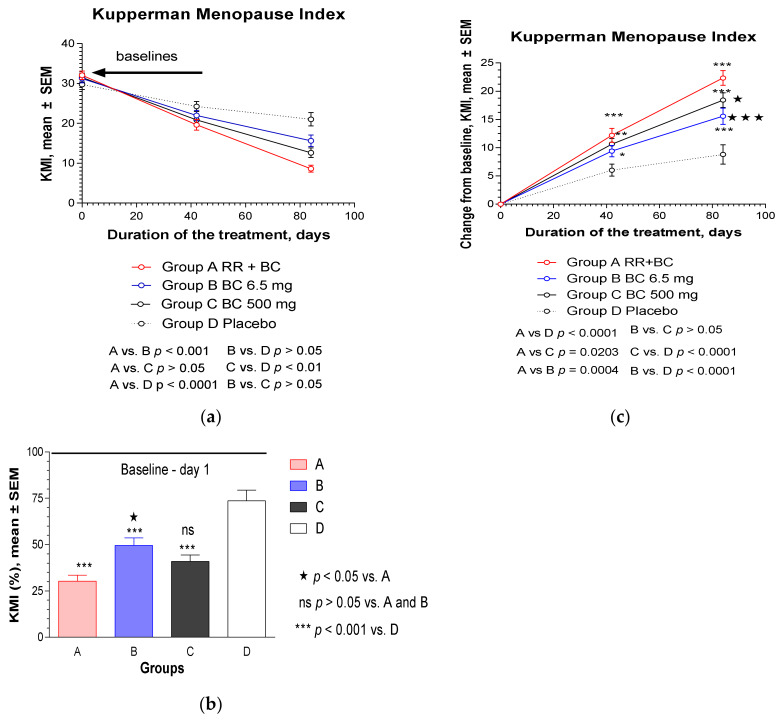
Effects of RR-BC (Group A), BC (Group B), BC500 (Group C), and placebo (Group D) on menopausal symptoms after 12 weeks (84 days) of treatment, as assessed using the KMI: (**a**) a significant decrease of KMI (mean ± SEM) score compared to baseline values was observed in all groups over time (*p* < 0.0001); (**b**) a significant difference in KMI (% to baseline, 100%, mean ± SEM) was observed in groups A, B, and C compared to placebo group at the end of the treatment—day 84, (*p* < 0.0001), as well as between groups A and B, but not in groups A vs. C and B vs. C; (**c**) statistically significant interaction effects between treatment groups and response (change from the baseline of KMI, mean ± SEM) over time showed significant difference between group A and groups B, C, and D, as well as groups B vs. C and B vs. D, but not B vs. D (two-way ANOVA). The significance of difference between groups at various time points, calculated by Bonferroni post-test, is expressed by symbols * *p* < 0.05, ** *p* < 0.01, *** *p* < 0.001, ns: not significant (A, B, and C vs. placebo); and * *p* < 0.05 (B vs. A), *** *p* < 0.001 (C vs. A).

**Figure A1:**
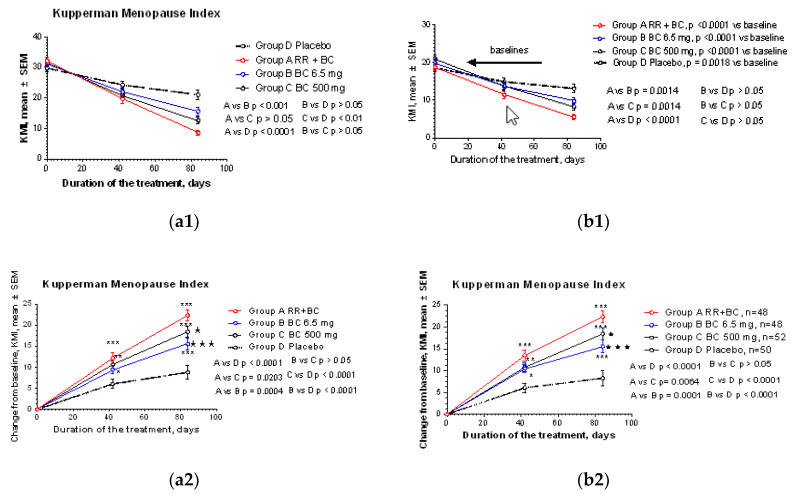
KMI (**a1**) -ITT analysis, (**b1**) -PP analysis. The change from baseline of KMI (mean ± SD) over time: (**a2**) -ITT analysis, (**b2**) -PP analysis. Significance of difference between groups at various time points, calculated by Bonferroni post-test, is expressed by symbols * *p* < 0.05, ** *p* < 0.01, *** *p* < 0.001; ns: not significant (A, B, C vs. placebo) and * *p* < 0.05 (C vs. A), *** *p* < 0.001 (B vs. A).

Compared to the baseline values, a significant decrease in KMI score was observed in all the study groups after repeated administration of the investigated product (IP) or placebo for 6 and 12 weeks (Figure 2a). Figure 2b shows the effect size (%) after 12 weeks of treatment, compared to the baseline values (100%). The strongest effect was observed in Group A, in which the patients were administered RR-BC (a reduction in KMI score by 71.2% from the baseline), while for the BC, BC500, and placebo groups, the values were 50.5%, 59.1%, and 26.3%, respectively. Effects in all treatment groups were significantly higher (*p* < 0.0001) than the placebo group. The effect of RR-BC was significantly better than the effect of BC (*p* < 0.05). The changes in KMI scores from the baseline over time are shown in Figure 2c and in Figure A1. The overall effects of RR-BC were superior to the effects of BC (*p* < 0.001, two-way ANOVA) and BC500 (*p* < 0.05, two-way ANOVA) over time (Table A2 in Appendix A). There was no significant difference between the effects of BC and BC500 over time (Table A2 in Appendix A).

**Figure 3:**
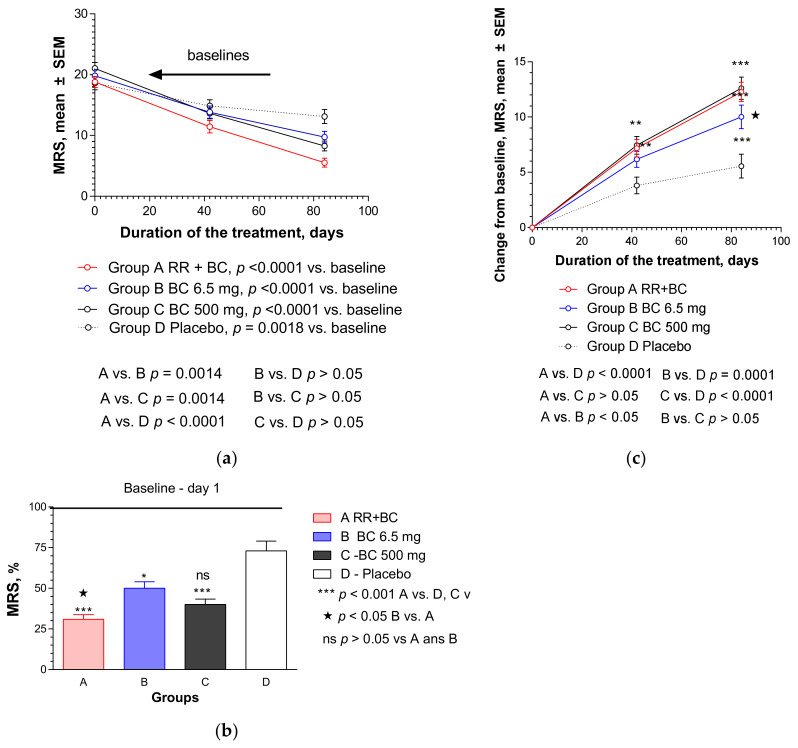
Effects of RR-BC (Group A), BC (Group B), BC500 (Group C), and placebo (Group D) on menopausal symptoms after 12 weeks (84 days) of treatment, as assessed using MRS: (**a**) significant decrease of MRS (mean ± SEM) score compared to baseline values was observed in all the groups over time (*p* < 0.0001 in groups A, B, C; *p* = 0.0018—in placebo group); (**b**) significant difference in MRS (% to baseline, 100%, mean ± SEM) was observed in A, B, and C groups compared to placebo group at the end of the treatment—day 84, (*p* < 0.001, A vs. D and B vs. D), as well as between groups A and B, but not groups A vs. C and B vs. C; (**c**) statistically significant interaction effects between treatment groups and response (change from the baseline of MRS, mean ± SEM) over time showed significant differences between groups A, B, and C vs. placebo, as well as groups B vs. C, but not A vs. B (two-way ANOVA). Significance of difference between groups at various time points, calculated by Bonferroni post-test, is expressed by symbols * *p* < 0.05, ** *p* < 0.01, *** *p* < 0.001; ns: not significant (A, B, and C vs. placebo) and * *p* < 0.05 (B vs. C).

Similar results were obtained when using MRS to assess menopause severity, which includes the assessment of urogenital symptoms (sexual problems, bladder problems, vaginal dryness), in addition to somatic and psychological symptoms measured by KMI (Figure 3).

A significant decrease in MRS scores compared to the baseline values was observed in all study groups after repeated administration of IPs or placebo for 6 and 12 weeks (Figure 3a). Figure 3b shows the effect sizes (%) after 12 weeks of treatments compared to the baseline values (100%). The strongest effect was observed in Group A, in which the patients were administered the RR-BC combination; the reduction of MRS was 67.7% from the baseline, while in the BC, BC500, and placebo groups, it was 49.9%, 60.0%, and 26.9%, respectively. Effects in treatment groups were significantly higher (*p* < 0.05 in BC and *p* < 0.0001 in RR-BC and BC500) compared to the placebo group (Figure 3b).

**Figure A2:**
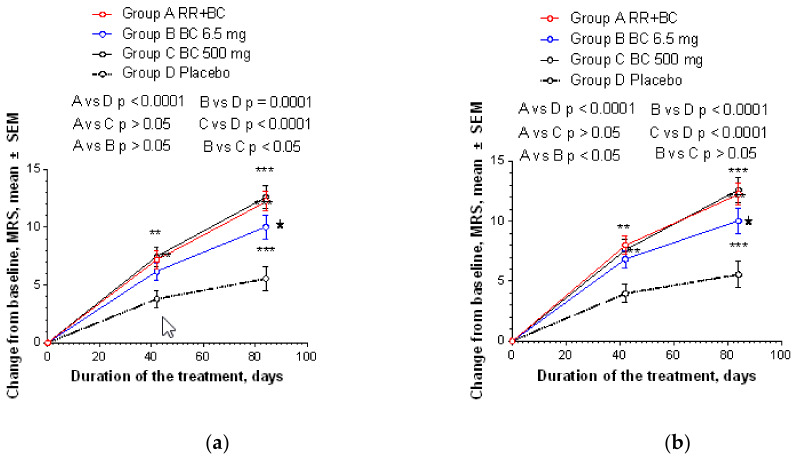
The change from baseline of MRS (mean ± SD) over time: (**a**) -ITT analysis, (**b)** -PP analysis. Significance of difference between groups at various time points, calculated by Bonferroni post-test, is expressed by symbols ** *p* < 0.01, *** *p* < 0.001; ns: not significant (A, B, C vs. placebo) and * *p* < 0.05 (B and C vs. A).

The effect of RR-BC was significantly greater than the effect of BC (*p* < 0.05) at the end of the treatment (Day 84). The changes in MRS from the baseline over time are shown in Figure 3c and in Appendix A (Figure A2 and Table A3). The effects of RR-BC, BC, and BC500 were superior to the effects of placebo (*p* < 0.0001, two-way ANOVA) over time, and particularly at the end of the 6-week treatment course (Table A3b). There was no significant difference between the effects of RR-BC and BC or BC500 over time (*p* > 0.05, two-way ANOVA, Table A3a), while the effect of BC500 was superior compared to BC (*p* < 0.05, Table A3a – ITT analysis). However, there was a significant difference between the effects of RR-BC and BC over time (*p* < 0.05, two-way ANOVA), and no significant differences in the effects of BC and BC500 (*p* < 0.05) in the subset of 198 patients who have completed all tests per protocol (PP) (Table A3b in Appendix A).

#### 2.2.2. Secondary Efficacy Endpoint

**Figure 4:**
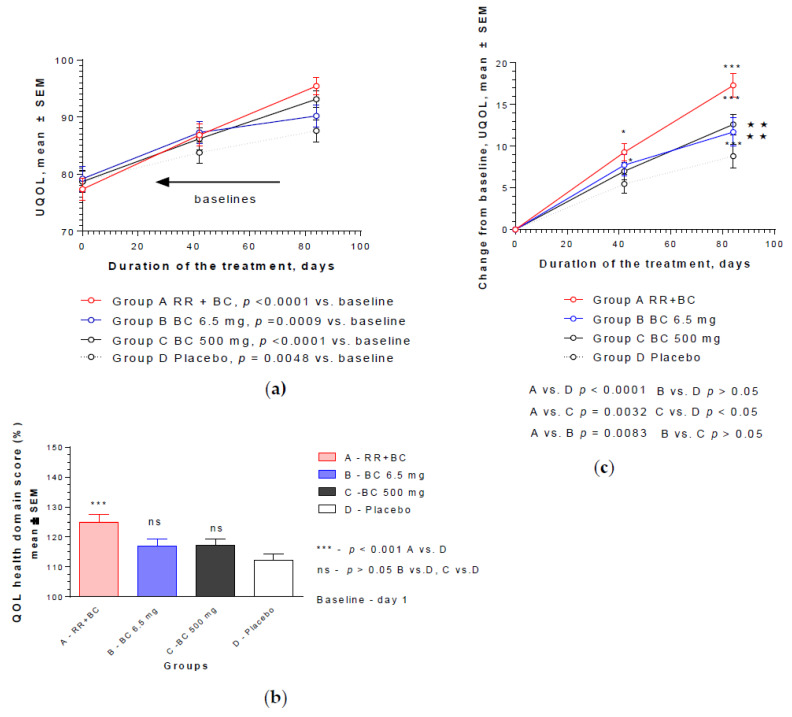
Effects of RR-BC (Group A), BC (Group B), BC500 (Group C), and placebo (Group D) on overall quality of life after 12 weeks (84 days) of treatment, as assessed by the UQOL scale: (**a**) significant increase of UQOL (mean ± SEM) score compared to baseline values was observed in all groups over time (*p* < 0.01; *p* < 0.001); (**b**) significant difference in UQOL (% to baseline, 100%, mean ± SEM) was observed in group A compared to placebo group at the end of the treatment—Day 84, (*p* < 0.0001), but not groups B or C vs. placebo; (**c**) statistically significant interaction effects between treatment groups and response (change from the baseline of UQOL, mean ± SEM) over time showed significant difference between groups A, B, and C vs. placebo, as well as group A vs. groups B and C (*p* = 0.0083 and *p* = 0.032) but not between the two BC groups, B and C (two-way ANOVA). Significance of difference between groups at various time points, calculated by Bonferroni post-test, is expressed by symbols * *p* < 0.05, *** *p* < 0.001; ns: not significant (A, B, C vs. placebo) and ** *p* < 0.01 (B and C vs. A).

Compared to the baseline values, all four preparations significantly increased the QOL of patients as measured by UQOL questionnaire after 6 and 12 weeks of daily treatment (Figure 4a). The most significant difference compared to the baseline was observed in the RR-BC and BC500 groups (*p* < 0.0001). Figure 4b shows the effect size (%) after 12 weeks of treatment, compared to the baseline values (100%).

The strongest effect was observed in Group A, in which the patients were administered the RR-BC combination: the increase of QOL score was 25% from the baseline, while in the BC, BC500, and placebo groups it was 17.0%, 17.2%, and 12.1%, respectively. The only statistically significant difference was observed in the RR-BC group (*p* < 0.0001) compared to the placebo effect, while the differences between BC, BC500, and placebo were insignificant (*p* > 0.05). The changes in QOL from the baseline over time are shown in Figure 4c; the overall effects of RR-BC were superior to the effects of BC (*p* = 0.0083, two way ANOVA) and BC500 (*p* = 0.0032, two way ANOVA) over time, and particularly at the end of the 12 week treatment course. There was no significant difference between the effects of BC and BC500 over time (*p* > 0.5).

**Figure 5:**
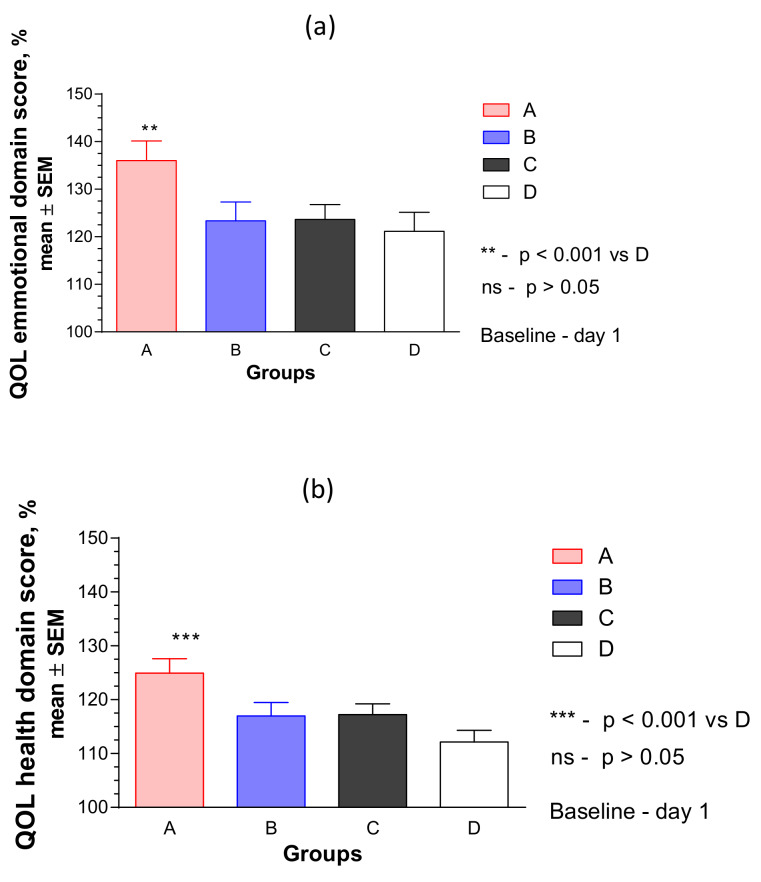
Effects of RR-BC (Group A), BC (Group B), BC500 (Group C), and placebo (Group D) on (**a**) emotional and (**b**) physical health domains of quality of life after 12 weeks of treatment, as assessed by the UQOL scale. A significant difference in UQOL (% to baseline, 100%, mean ± SEM) was observed in group A compared to placebo group at the end of the treatment—Day 84, (*p* < 0.0001), but not between groups B or C vs. placebo; ns: not significant.

A detailed analysis of these effects on the four QOL domains revealed no substantial effect of treatment on the social and environmental domains, while the most pronounced effects were observed in the health and emotional domains, where the effect size of QOL score increased by 25% and 36%, respectively, compared to the baseline (Figure 5).

Statistically significant interaction effects between treatment groups and response (change from the baseline of emotional UQOL) over time showed a significant difference between groups A vs. B, C, and placebo groups (respectively, *p* = 0.0187, *p* = 0.0013, *p* < 0.001), and particularly at the end of the 12 week treatment course, Figure 5a. However, there was no significant difference between the effects of BC, BC500, and placebo over time (*p* > 0.5).

Figure 5b shows a significant difference in the effects of treatment groups A, B, and C vs. placebo (respectively, *p* < 0.0001, *p* = 0006, *p* < 0.05), as well as group A vs. group C (*p* < 0.001), but not between the two BC groups (BC and BC500, two-way ANOVA) on the physical health domain of UQOL. The overall effects of RR-BC were superior to the effect of BC500 (*p* = 0.0026, two-way ANOVA), and predominantly at the end of the 12-week treatment course.

**Figure 6:**
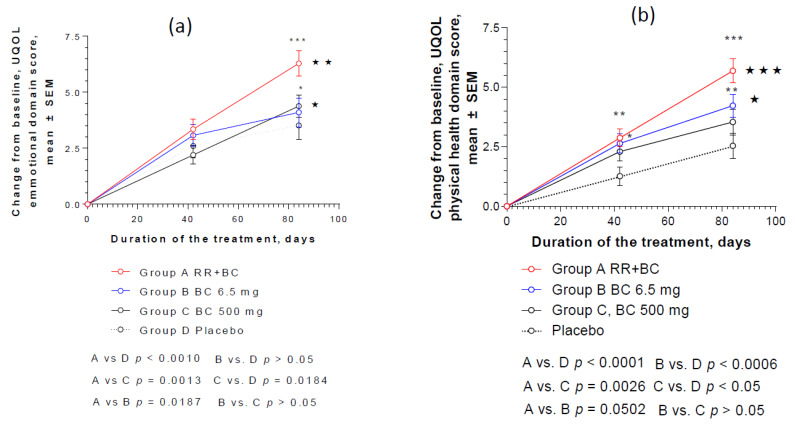
Effects of RR-BC (Group A), BC (Group B), BC500 (Group C), and placebo (Group D) on (**a**) emotional and (**b**) physical health domains of quality of life after 12 weeks (84 days) of treatment, as assessed by the UQOL scale: (**a**) statistically significant interaction effects between treatment groups and response (change from the baseline of physical UQOL, mean ± SEM) over time showed significant difference between groups A, B, and C vs. placebo (respectively *p* < 0.0001, *p* = 0006, *p* < 0.05), as well as group A vs. group C (*p* < 0.001), but not the two BC groups, B and C (two-way ANOVA); (**b**) statistically significant interaction effects between treatment groups and response (change from the baseline of emotional UQOL, mean ± SEM) over time showed significant difference between groups A vs. B, C, and placebo groups (respectively, *p* = 0.0187; *p* = 0.0013; *p* < 0.001). Significance of difference between groups at various time points, calculated by Bonferroni post-test, is expressed by symbols * *p* < 0.05, ** *p* < 0.01, *** *p* < 0.001, ns: not significant (A, B, C vs. placebo); and * *p* < 0.05, ** *p* < 0.01 *** *p* < 0.001 (A vs. B and C).

The results of the efficacy analysis on a subset of 198 patients who completed the study (PP) were mainly in line with the results obtained in 220 patients (ITT), including the 22 dropouts (Appendix A), except for the significant improvement in sexual activity in the RR-BC group compared to the placebo (Table 2b and Figure 6).

### 2.3. Safety Evaluation

#### 2.3.1. Extent of Exposure

There was no significant difference between groups in terms of treatment duration and the extent of exposure (Supplementary material 1). The mean duration of treatment in the study groups was 10.32 weeks (median: 11 weeks). No significant difference was observed in time to treatment failure (TTF)/dropout (Supplementary materials 1 and 2), indicating that the study groups were comparable throughout the whole study period, which is important for the successful assessment of efficacy and safety.

#### 2.3.2. Adverse Events

Adverse events (AE), regardless of causality, were recorded for all patients after 6 and 12 weeks of treatment. An overall summary of the AEs observed in this study is presented in Table 3 and Supplementary materials 1 and 2.

A total of 21 patients (9.5%) enrolled in this study reported AEs (Table 3 and Supplementary materials 1 and 2). The number of AEs in the treatment groups (*n* = 6, 21% of all AEs) was lower than in the placebo group (*n* = 11, 38% of all AEs), although the difference was not statistically significant (Table 3). The number of patients who experienced AEs was similar in all of the treatment groups.

The list of AEs observed in this trial and their distribution between the study groups are shown in Table 4 and Supplementary materials 1 and 2.

The types of AE were similar in all the groups, with the maximum number of AEs reported in the placebo group. Statistical testing did not reveal any significant difference between the groups with regard to the type, frequency, or severity of AEs. The most frequent AE was gastrointestinal pain, which was recorded in nine patients (4.1%, less than the commonly accepted limit of statistical confidence of 5.0%). These results suggest that none of the AEs were related to the IPs. Furthermore, BC was safe to use at a dose of 500 mg. All of the AEs were of Grade 1–2 severity and none were considered to be related to the treatment. No serious AE was recorded in this study.

## 3. Discussion

The clinical efficacy of BC for treating menopause syndromes has been demonstrated in many clinical studies; however, the number of dose–effect comparative studies to establish the most effective dose of a compound has been limited. In a recent randomized, double-blind, placebo-controlled trial in patients with climacteric symptoms, dose-dependent improvements in symptom severity and quality of life (QOL) were seen after treatment with two doses of the BC extract Ze 450 (6.5 mg and 13 mg daily) over 12 weeks, especially in patients with severe symptoms 11 [8]. Furthermore, in another observational study by Drewe et al. in 2013 [12], treatment with BC in unselected patients with climacteric complaints, under the conditions of daily practice, resulted in a significant improvement of menopausal symptoms, as evaluated by the total Kupperman Menopausal Index (KMI) score and its subitem scores [26]. The effect size observed in this study was similar to that observed in a previous randomized, controlled clinical trial.

The objective of this randomized controlled study was to compare the efficacy and safety of low- and high-dose BC treatment with the efficacy and safety of a BC-RR hybrid combination (Menopause Relief EP^®^) in treating menopausal syndrome.

ITT analysis showed that the treatment effect was in favor of the BC-RR combination. The superior effect of BC-RR versus placebo in relief of menopausal symptoms (measured via KMI [28] and MRS [29,30] scores) was observed after 6 weeks of treatment, while a superior effect compared to BC and BC500 was observed after 12 weeks of treatment (Figure 2 and Figure 3) in both the patients who completed the treatment and those who were included in the ITT analysis.

The positive response to the high dose of BC (daily dose, 1000 mg BC500) was slightly higher than to the normal daily dose of 13 mg (two capsules of BC 6.5 mg); however, the difference was not statistically significant (Figure 2 and Figure 3). There was no substantial difference in the safety of these two doses, as assessed by incidence and severity of emergent AEs (Table 3 and Table 4).

**Figure A6:**
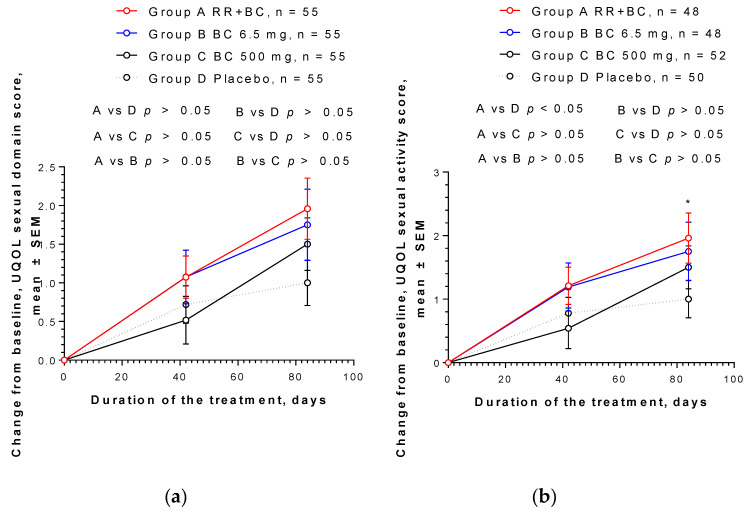
The change from baseline of menopause UQOL sexual domain scores (mean ± SD) over time: (**a**) -ITT analysis, (**b**) -PP analysis. Significance of difference between groups at various time points, calculated by Bonferroni post-test, is expressed by symbols * *p* < 0.05, ns: not significant (A, B, C vs. placebo).

Significant improvement was observed in QOL of all patients, particularly in the group treated with the hybrid combination of Menopause Relief EP^®^ (Figure 5 and Figure 6). The difference in the improvement of QOL score was significantly higher compared to the effect of BC, BC500, or placebo, particularly in the emotional domain of QOL and specifically related to the sexual activity of patients (Table A7b and Figure A6b).

These observations suggest a contribution of *Rhodiola* extract to the overall effect of the RR-BC combination in improving the QOL of the patients. The improvement in QOL was observed only in the emotional and physical health domains of QOL (Figure 5 and Figure 6), but not in the social and environmental domains (Supplementary material 2). This observation is in line with several previous studies on the adaptogenic activity of *Rhodiola* and its beneficial effect on stress, as well as mental, behavioral, and aging-related disorders [30,35,36,37,38,41,42,43,44,45,46].

Further studies in which the clinical efficacy of BC-RR and RR is compared would provide an indication of whether RR is effective in the relief of menopausal symptoms, or whether these two plants work better as a hybrid combination. In other words, whether *Rhodiola* potentiates the effect of BC or both plants work synergistically remains unknown. Nonetheless, it is obvious that both plants work better together than BC alone.

All preparations were well tolerated, and the incidence characteristics of AEs suggest that none were related to the treatment. No serious AEs were recorded in the study.

## 4. Materials and Methods

### 4.1. Study Design

**Figure 7:**
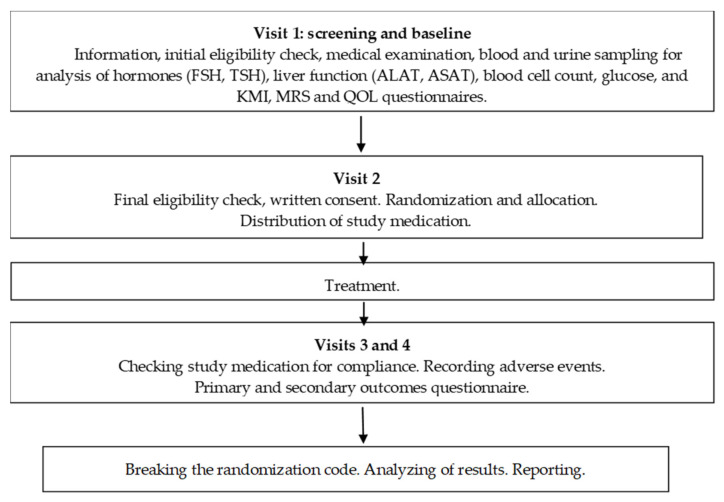
Schematic diagram of the trial.

This was a phase II, four-arm parallel group, placebo-controlled, randomized, double-blind, single-institution clinical trial designed to investigate the efficacy and safety of the fixed combination of *Cimicifuga* EP-40 and *Rhodiola* EPR-7 extracts (Menopause Relief EP^®^, RR-BC) versus *Cimicifuga* EP-40 (BC) therapy alone in adult women with menopausal complaints. Eligible patients were randomized to one of the four study groups: RR-BC, BC, BC500, or placebo. The treatments were orally administered once per day for 12 consecutive weeks. The schedule of examinations and procedures evaluations included four visits: 1 week before treatment, at the beginning of the treatment, and at 6 and 12 weeks after treatment (Figure 7 and Table 5).

The study was carried out at the I. Zhordania Institute of Reproductology, Head Professor Archil Khomasuridze, Tbilisi, Georgia, with the approval of the Ethical Committee Board (ERB) (Registration Nr 02-18, date of approval of final protocol 15 February 2018). ClinicalTrials.gov Identifier: NCT03461380 [47].

All patients provided written informed consent to participate in the study prior to inclusion. The information about the study was provided to the study participants in both Georgian and English languages, in accordance with local regulations. The patient information sheet described the study procedures, aims, potential risks, and expected benefits. The investigator explained the content of the document to each patient in detail. The patients had time to consider the information before signing the informed consent form in order to confirm that they fully understood the information and willingly volunteered to participate in the study. Each patient was given a copy of the informed consent form, and the original copy was kept in a confidential file along with the case report form (CRF).

### 4.2. Selection of the Study Population

#### 4.2.1. Inclusion and Exclusion Criteria

The target population for this study consisted of female patients aged 40 to 82 years (mean age: 52.5 ± 7.7 years), with the diagnosis of “menopausal female climacteric states” (N95.1 according to the International Statistical Classification of Diseases and Related Health Problems 10th Revision, ICD-10, Version for 2014 [48]) and confirmed by high blood levels of follicle-stimulating hormone (FSH), low levels of estrogen (estradiol, E2), and normal level of thyroid-stimulating hormone (TSH), Supplementary material 2. Some patients were receiving thyroxin due to hypothyroidism; however, at the time of enrolment, they had normal thyroid function. Eligible patients had anamnestically stable menopausal symptoms such as flushing, sleeplessness, headache, and lack of concentration observed during at least the previous 2 weeks. They had moderate KMI scores (20–34, mean value: 31.1 ± 8.5), moderate Menopause Rating Scale scores (MRS, mean value: 19.6 ± 7.6), and QOL scores of 78.5 ± 14.7 as estimated at baseline (Visit 1) by the clinician responsible.

Subjects with previous or current psychological disorders that could interfere with their ability to participate in the study were excluded. Other exclusion criteria were as follows: anamnestic or current alcohol or drug abuse, concomitant treatment with psychotropic drugs (in particular, benzodiazepines, antidepressants, hypnotics, or neuroleptics) or hormonally acting drugs (such as hormone replacement therapy), hyperthyroidism, malignant tumors, continuous climacteric bleeding and complaints related to myomas, patients who had taken another experimental drug within a 4 week period prior to the trial, pregnancy/lactation, serious internal disease, previous organ transplantation, hypersensitivity to one of the ingredients of the trial medication, or a body mass index higher than 30. Women using contraceptive pills or concomitant treatment with other herbals or food supplements were excluded. During the study, the patients did not receive any medication that might influence the outcome measures.

#### 4.2.2. Recruitment and Screening

Individuals were recruited by doctors at the Clinic of I. Zhordania Institute of Reproductology, Head Professor Archil Khomasuridze, Tbilisi, Georgia, in the course of attendance of patients to the clinics. The screening procedures for eligibility to participate in the study were applied after receiving a voluntary written informed consent. All applicable principles of the Declaration of Helsinki, the ICH guidelines, and EMEA clinical trials guidelines were considered. In the course of the patients’ initial visit to the study site, inclusion and exclusion criteria were checked against the eligibility checklist and individuals interested in participating received relevant information about the study. Patients were initially given a physical examination performed by an investigator. Relevant lab tests and specialist consultations were arranged if necessary. When inclusion criteria were met, patients consented to participate the study and underwent randomization.

#### 4.2.3. Participant Withdrawal

Participants were free to withdraw from the study at any time without giving a reason, and with no negative consequences. There were 22 cases of participant dropouts from the study.

#### 4.2.4. Ethics Approval and Consent to Participate

Ethical approval was obtained from the I. Zhordania Institute of Reproductology, Head Professor Archil Khomasuridze, Tbilisi, Georgia, with the approval of the Ethical Committee Board (ERB) (Registration Nr 02-18, date of approval of final protocol 2018-02-15). The study was conducted in accordance with the principles of Good Clinical Practice, according to the International Conference on Harmonization Guidelines. Ethics review procedures were conducted according to the Declaration of Helsinki and local laws and regulations. Written informed consent was obtained from all participants before study-specific procedures were performed.

### 4.3. Intervention and Comparator

Menopause Relief EP^®^ capsules, 206.5 mg (EuroPharma USA Inc., Green Bay, WI, USA) were manufactured according to ICH Guidelines for Good Manufacturing Practice (GMP). One capsule contained 200 mg of *R. rosea* Radix and rhizoma dry native extract EPR-7^®^ [40], (DER 2.5–5.0:1, extraction solvent—70% ethanol), and 6.5 mg of *C. racemosa* (L.) Nutt., rhizoma dry extract, EP-40^®^, (DER 4.5–8.5:1, extraction solvent—60% ethanol), and inactive excipients, microcrystalline cellulose and magnesium stearate. The placebo capsules (containing 600 mg of microcrystalline cellulose and magnesium stearate) and capsules containing 6.5 mg (BC) and 500 mg (BC500) of the same BC rhizoma extract and inactive excipients (microcrystalline cellulose, magnesium stearate) were manufactured according to ICH Guidelines for GMP. The appearance, smell, and color of all preparations was similar, and they were organoleptically indistinguishable. The products were packed, blinded, and labeled with the product name, study code, and storage conditions. The investigational products (IPs) were packed in HDPE bottles, each containing 180 capsules, of which 168 capsules were used during the scheduled 84 days and 12 extra capsules were used for the 6-day grace period. Each patient received one bottle on Visit 2 (Day 1).

Herbal preparations were qualitatively and quantitatively tested by HPLC (Supplementary material 1) in accordance with specifications, using reference standards. All analytical methods were validated for selectivity, accuracy, and precision. Reference samples were retained and stored at QC EuroPharma USA Inc. (Green Bay, WI, USA).

All study products were kept in a secure place under appropriate storage conditions. A description of the appropriate storage and shipment conditions was specified on the investigational product label and investigator brochure. The storage was locked and only accessible to authorized personnel.

#### 4.3.1. Doses and Treatment Regimens

The preparations (two capsules) were orally administered once per day for 84 consecutive days. The investigator was responsible for maintaining drug accountability records for the study products. Drug accountability for this study was ensured in accordance with the standard procedures.

#### 4.3.2. Randomization and Blinding

Study preparations (packages of placebo and verum capsules) were labeled by a qualified pharmacist (QP) at the manufacturing site using the random number generator in Microsoft Excel. The randomization sequence contained information for encoding RR-BC, BC, BC500, and placebo capsules in four columns (A, B, C, and D, respectively) filled with randomly distributed unique numbers (treatment code no.) from 1 to 220.

#### 4.3.3. Allocation Concealment

The random sequences of the treatments were kept confidential by the QP at the investigational product manufacturing site (at sponsor) until the study was finalized. These were provided to the principal investigator before statistical evaluation of the results, when all the patients had completed the treatment.

#### 4.3.4. Implementation and Blinding

At Visit 2, all participants received a consecutive number ranging from 001 to 220. Participants were sequentially enrolled by the principal investigator. Each patient was also assigned a random treatment code and received the capsules in the corresponding package. The patient allocation sequence (participant list) identifying the patients and study supplement packages was generated and maintained by the principal investigator, who filled out the patient names in the CRF and on the package label.

Blinding for trial subjects was performed via the use of labeled packages containing vials with capsules of identical appearance. Study preparations were delivered to the clinic pre-labeled and coded according to the randomization list. The randomization code was kept secret from the clinic and the participating investigators and only revealed after termination of the study. In this way, the investigators were also blinded to the study medication and placebo control, ensuring a double-blind design. Information concerning the allocation of participants was also kept in sequentially numbered and sealed envelopes that were stored by the qualified pharmacist of the manufacturer organization. Individual treatment codes, indicating treatment randomization for each randomized participant, were available to the investigator and the sponsor in such a format that they could de-blind individuals or the whole group, should it be necessary. However, there were no cases in which this occurred.

#### 4.3.5. Evaluation of Compliance

To ensure drug accountability, the investigator maintained accurate records of the dates and amounts of drug received, to whom it was dispensed (Supplementary materials 1 and 2). Treatment compliance was assessed by a capsule count of the returned medication. Overall compliance in each group was more than 90% comprising 92.75 ± 2.75, 90.13 ± 3.52, 97.15 ± 1.54, and 93.33 ± 2.86 in groups A, B, C, and D, respectively. There was no significant deference between groups (Supplementary materials 1 and 2).

Treatment compliance was ensured by monitoring the records and analysis by treatment group and time interval. The study investigators checked overall compliance with the study protocol at each visit, and the remaining capsules were counted at the end of the study. All unused capsules and the drug accountability forms were recorded and returned at the end of the study to the sponsor. The reference samples of the batch used in this study were retained in the archive storage of the sponsor.

### 4.4. Efficacy and Safety Outcomes

The primary efficacy outcome measures of the study were the relief of menopausal symptoms, measured using the total KMI [26] and MRS [27,28] scales (Appendix A, Table A1a), including:

- Somatic symptoms (hot flushes, sweating, heart discomfort, sleep problems, and joint and muscular discomfort);
- Psychological symptoms (depressive mood, irritability, anxiety, physical, and mental exhaustion);
- Urogenital symptoms (sexual problems, bladder problems, and dryness of vagina).

The secondary efficacy endpoint of the study was improvement in menopause QOL, assessed by the validated Utian Quality of Life (UQOL) scale [29].

Safety and tolerability were assessed by monitoring the frequency, duration, and severity of AEs (Supplementary material 1), evaluating withdrawal/time to treatment failure (TTF) (Supplementary material 1), and via hematological and biochemical blood parameters. Assessments included laboratory tests, such as TSH, FSH, E2, ALAT, ASAT, blood urea, and creatinine, and urine microscopy and urine pregnancy tests.

### 4.5. Efficacy and Safety Evaluation

#### 4.5.1. Efficacy Primary Endpoint

Evaluation of the primary efficacy endpoints was archived by intergroup comparison (verum vs. placebo) of the changes of KMI and MRS scores from the baseline (Day 0) to the end of the 12-week therapy.

#### 4.5.2. Efficacy Secondary Endpoints

Evaluation of the secondary efficacy endpoint was archived by intergroup comparison (verum vs. placebo) of the changes of UQOL score from the baseline (Day 0) to the end of the 12-week therapy.

#### 4.5.3. Safety Outcomes

Safety and tolerability were assessed by monitoring the frequency, duration, and severity of AEs (Supplementary materials 1 and 2), evaluating withdrawal/time to treatment failure (TTF) (Supplementary material 1), and via hematological and biochemical blood parameters. Assessments included laboratory tests, such as TSH, FSH, E2, blood urea, and creatinine, and urine microscopy and urine pregnancy tests.

### 4.6. Statistical Analysis

The clinical data at each visit were recorded using a standardized clinician assessment form (Supplementary material 1). Edited and corrected data were added to an Excel database (Supplementary material 2) that was input to Prism statistical software (version 3.03 for Windows; GraphPad, San Diego, CA, USA). The change in scores of MRS, KMI, and UQOL from the initial visit (baseline) to intermediate and final visits, and at each scheduled visit during the study, were measured.

The primary population was the intent-to-treat (ITT) population, which was defined as all randomly assigned patients who received at least one dose of the study medication. Statistical analysis was performed using “observed” data on an ITT basis for time-to-event outcomes.

Baseline characteristics were compared using tools for assessing column statistics and the KW non-parametric one-way ANOVA rank order test with a post hoc Dunn’s multiple comparison test to compare the four groups. The same statistical tools were used to analyze changes within treatment groups over time (ITT analysis). Additional statistical analysis was performed for patients who completed the treatment and passed all tests in all visits to the clinic. Within-group repeated measures analysis of variables was performed with one-way, repeated-measures ANOVA (data with normal distribution) or the Friedman non-parametric rank test.

The efficacy of the study preparations was assessed by two-way between–within ANOVAs, in which an interaction effect indicates a different response over time between the two groups and would therefore signal a treatment effect.

The incidence of AEs was compared across treatment groups for descriptive purposes and to identify possible differences in the safety profiles using two methods for categorical data.

#### Sample Size Considerations

Based on KMI data from previous clinical trials, the sample size was conservatively estimated as *n* = 60 patients/group. Assuming an alpha-error = 0.05, mean KMI scores (standard deviation, SD) of 27 (15) and 18 (15) in the placebo and verum groups, respectively, and an anticipated 10% drop-out rate, and using the Kruskal–Wallis (KW) non-parametric one-way analysis of variance (ANOVA) rank order test with post hoc Dunn’s multiple comparison test, a power (1−β) of > 90% could be expected.

We planned to enroll 220 patients in this study. The proposed sample size would have a power of 90% to detect an effect size of 0.6, with a two-sided significance level of 0.05. We required 50 patients in each group and, assuming a dropout rate of 10%, we intended to recruit 55 patients for each group. The effect size was calculated by dividing the expected rate of reduction of KMI, taken as 17 with an SD of 9 [11].

## 5. Conclusions

In this four-arm parallel group, placebo-controlled, randomized, double-blind comparative clinical study, we demonstrated for the first time that the black cohosh–rose root (BC-RR) extracts hybrid combination (Menopause Relief EP^®^) was significantly superior to preparations of black cohosh in relief of menopausal symptoms, regardless of the dose of BC. Furthermore, unlike BC, Menopause Relief EP^®^ significantly improved menopausal QOL, mainly due to its superior effect on emotional and physical health domains, apparently due to the contribution of RR extract to the overall effect of the hybrid combination of BC-RR. The difference in the effects of high and normal doses of BC was not significantly different.

## Figures and Tables

**Table 1:** (**a**) Baseline demographic and efficacy outcome measures; (**b**) baseline characteristics of patients who completed the treatment per protocol (PP).

(**a**)							
	**Mean ± SD**				**Mean Difference and *p*-Value**		
	**A**	**B**	**C**	**Placebo**	**A vs. PL**	**B vs. PL**	**C vs. PL**
	**(*n* = 55)**	**(*n* = 55)**	**(*n* = 55)**	**(*n* = 55)**			
Age (years)	52.36 ± 7.30	52.60 ± 8.07	52.56 ± 8.58	52.53 ± 6.75	−0.164	0.073	0.036
					*p* > 0.05 ^ns^	*p* > 0.05 ^ns^	*p* > 0.05 ^ns^
BMI (kg/m^2^)	25.71 ± 2.63	26.08 ± 3.42	26.23 ± 2.80	25.97 ± 3.22	−0.255	0.109	0.262
					*p* > 0.05 ^ns^	*p* > 0.05 ^ns^	*p* > 0.05 ^ns^
MRS	18.78 ± 6.65	19.82 ± 7.96	21.05 ± 7.31	18.60 ± 8.22	0.1818	−1.236	2.455
					*p* > 0.05 ^ns^	*p* > 0.05 ^ns^	*p* > 0.05 ^ns^
KMI	32.07 ± 7.99	31.25 ± 8.249	31.51 ± 8.366	29.76 ± 9.50	2.309	1.491	1.745
					*p* > 0.05 ^ns^	*p* > 0.05 ^ns^	*p* > 0.05 ^ns^
UOQL	77.36 ± 14.50	79.18 ± 16.60	78.69 ± 14.49	78.73 ± 13.17	−1.364	0.454	−0.036
					*p* > 0.05 ^ns^	*p* > 0.05 ^ns^	*p* > 0.05 ^ns^
(**b**)							
	**Mean ± SD**				**Mean Difference and *p*-Value**		
	**A**	**B**	**C**	**Placebo**	**A vs. PL**	**B vs. PL**	**C vs. PL**
	**(*n* = 48)**	**(*n* = 48)**	**(*n* = 52)**	**(*n* = 50)**			
Age (years)	52.36 ± 7.30	52.60 ± 8.07	52.56 ± 8.58	52.53 ± 6.75	−0.164	0.073	0.036
					*p* > 0.05 ^ns^	*p* > 0.05 ^ns^	*p* > 0.05 ^ns^
BMI (kg/m^2^)	25.71 ± 2.63	26.08 ± 3.42	26.23 ± 2.80	25.97 ± 3.22	−0.255	0.109	0.262
					*p* > 0.05 ^ns^	*p* > 0.05 ^ns^	*p* > 0.05 ^ns^
MRS	17.79 ± 6.39	19.75 ± 7.96	20.85 ± 7.35	18.78 ± 8.56	−0.984	0.975	2.17
					*p* > 0.05 ^ns^	*p* > 0.05 ^ns^	*p* > 0.05 ^ns^
KMI	30.98 ± 7.76	31.19 ± 8.42	31.04 ± 8.26	29.90 ± 9.59	1.313	1.521	1.138
					*p* > 0.05 ^ns^	*p* > 0.05 ^ns^	*p* > 0.05 ^ns^
UOQL	78.39 ± 14.78	78.77 ± 15.71	80.48 ± 12.68	78.78 ± 13.31	−0.117 > 0.05 ^ns^	0.266	1.701
						*p* > 0.05 ^ns^	*p* > 0.05 ^ns^

Abbreviations: SD: standard deviation; PL: placebo; BMI: body mass index (weight/height^2^); MRS: Menopause Rating Scale; KMI: Kupperman Menopausal Index; UQOL: Utian Quality of Life; ns – not significant.

**Table 2:** (**a**) The change from baseline of UQOL sexual activity score (mean ± SD) over time in all patients including dropouts. (**b**) The change from baseline of UQOL sexual activity score (mean ± SD) over time. Patients completed the treatment and all tests, dropouts excluded.

(**a**)										
**Time after Treatment**	**A**	**B**	**C**	**Placebo**	**Difference and *p*-Value**					
	**(*n* = 55)**	**(*n* = 55)**	**(*n* = 55)**	**(*n* = 55)**	**A vs. PL**	**B vs. PL**	**C vs. PL**	**A vs. B**	**A vs. C**	**B vs. C**
Day 0	0	0	0	0	0	0	0	0	0	0
	*n* = 55	*n* = 55	*n* = 55	*n* = 55						
Day 42	1.07 ± 2.0	1.07 ± 2.53	0.52 ± 2.27	0.72 ± 1.76	0.202 > 0.05 ^ns^	0.355 > 0.05 ^ns^	−0.20 > 0.05 ^ns^	0.001 > 0.05 ^ns^	0.556 > 0.05 ^ns^	0.557 > 0.05 ^ns^
	*n* = 54	*n* = 53	*n* = 54	*n* = 53						
Day 84	1.96 ± 2.74	1.75 ± 3.19	1.50 ± 2.45	1.00 ± 2.08	0.50 > 0.05 ^ns^	0.75 > 0.05 ^ns^	0.50 > 0.05 ^ns^	0.21 > 0.05 ^ns^	0.46 > 0.05 ^ns^	-0.25 > 0.05 ^ns^
	*n* = 48	*n* = 48	*n* = 52	*n* = 50						
Two-way ANOVA, *p*					>0.05 ^ns^	>0.05 ^ns^	>0.05 ^ns^	>0.05 ^ns^	>0.05 ^ns^	>0.05 ^ns^
(**b**)										
**Time after Treatment**	**A**	**B**	**C**	**Placebo**	**Difference and *p*-Value**					
	**(*n* = 48)**	**(*n* = 48)**	**(*n* = 52)**	**(*n* = 50)**	**A vs. PL**	**B vs. PL**	**C vs. PL**	**A vs. B**	**A vs. C**	**B vs. C**
Day 0	0	0	0	0	0	0	0	0	0	0
Day 42	1.21 ± 2.03	1.19 ± 2.63	0.54 ± 2.31	0.78 ± 1.77	0.43 > 0.05 ^ns^	0.41 > 0.05 ^ns^	−0.24 > 0.05 ^ns^	0.02 > 0.05 ^ns^	0.67 > 0.05 ^ns^	0.65 > 0.05 ^ns^
Day 84	1.96 ± 2.74	1.75 ± 3.19	1.50 ± 2.45	1.00 ± 2.09	0.96 < 0.05 *	0.75 > 0.05 ^ns^	0.50 > 0.05 ^ns^	−0.21 > 0.05 ^ns^	0.46 > 0.05 ^ns^	0.25 > 0.05 ^ns^
Two-way ANOVA, *p*					0.027	0.101 ^ns^	0.667 ^ns^	0.766 ^ns^	0.097 ^ns^	0.232 ^ns^

^ns^—not signoficant, *—significance *p* < 0.05.

**Table 3:** Number of patients who experienced adverse events (AE) and the number of AEs in each treatment group.

Treatment Groups	Number of Subjects Who Experienced AEs	%	Number of Adverse Events	% of Total AEs	Odds Ratio (D/A) Significance Level, *p*-Value
A. Black cohosh 6.5 mg + Rhodiola, 200 mg	5 of 55	9.1	6 of 29	20.7	2.0417 (z = 1.302) *p* = 0.1930
B. Black cohosh 6.5 mg	5 of 55	9.1	6 of 29	20.7	
C. Black cohosh 500 mg	5 of 55	9.1	6 of 29	20.7	
D. Placebo	6 of 55	10.9	11 of 29	37.9	
Total	21 of 220	9.5	29	100	

Abbreviation: AE: adverse event.

**Table 4:** Emergent adverse events observed in each treatment group.

AE	Treatment Related	Disease Related	Group A (*n* = 55)		Group B (*n* = 55)		Group C (*n* = 55)		Group D (*n* = 55)	
			*n*	%	*n*	%	*n*	%	*n*	%
Hot flashes		Yes	2	3.6					3	
Sweating		Yes	1	1.8					1	
Tachycardia		Yes					1			
Sleep disturbance	Yes								1	
Gastrointestinal pain	Yes		2	3.6	3	5.4	2		2	
Nausea	Yes				1	1.8			1	
Vomiting	Yes				1	1.8			1	
Diarrhea							1			
Hypertension	Yes								1	
Headache	Yes		1	1.8						
Allergic reaction	Yes									
Increase of appetite	Yes						1			
Anxiety	Yes						1			
Skin rush/pruritus	Yes				1	1.8			1	
Total			6	10.9	6	10.9	6	10.9	11	

**Table 5:** Schedule of examinations and procedures.

	Visit 1	Visit 2	At Home	Visit 3	Visit 4
	Day -7 Screening Baseline	Day 0	Days 1–4	Day 42	Day 84
Eligibility check/Information	x				
Informed consent	x	x			
Clinical examination	x			x	x
Enrolment and allocation to IP		x			
Treatment			x	x	x
MRS score	x			x	x
KMI score	x			x	x
QOL score	x			x	x
TSH	x				
FSH	x				
E2	x				
Urinalysis	x				
IP accountability				x	x
AEs				x	x

Abbreviations: IP: investigational product; MRS: Menopause Rating Scale; KMI: Kupperman Menopausal Index; QOL: quality of life; TSH: thyroid-stimulating hormone; FSH: follicle-stimulating hormone; E2: estradiol; AE: adverse event; ALAT: alanine aminotransferase; ASAT: aspartate aminotransferase.

**Figure A3:**
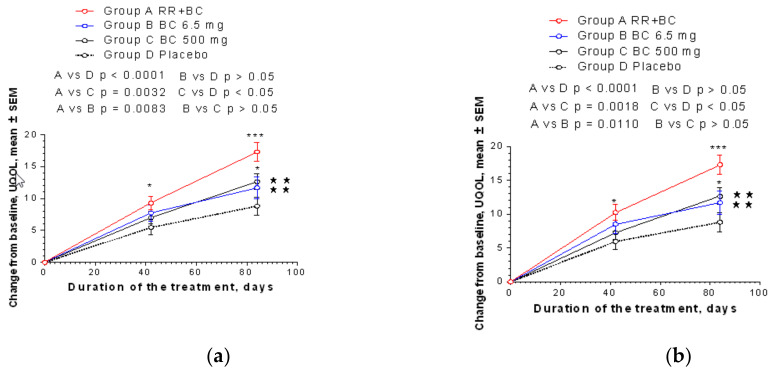
The change from baseline of menopause UQOL scores (mean ± SD) over time: (**a**) -ITT analysis, (**b**) -PP analysis. Significance of difference between groups at various time points, calculated by Bonferroni post-test, is expressed by symbols * *p* < 0.05, *** *p* < 0.001; ns: not significant (A, B, C vs. placebo) and ** *p* < 0.01 (B and C vs. A).

**Figure A4:**
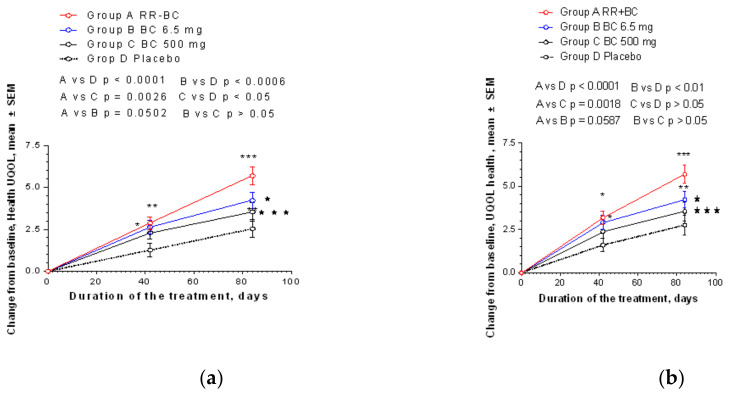
The change from baseline of menopause UQOL physical health domain scores (mean ± SD) over time: (**a**) -ITT analysis, (**b**) -PP analysis. Significance of difference between groups at various time points, calculated by Bonferroni post-test, is expressed by symbols * *p* < 0.05, ** *p* < 0.01, *** *p* < 0.001; ns: not significant (A, B, C vs. placebo) and * *p* < 0.05 (C vs. A), *** *p* < 0.001 (B vs. A).

**Figure A5:**
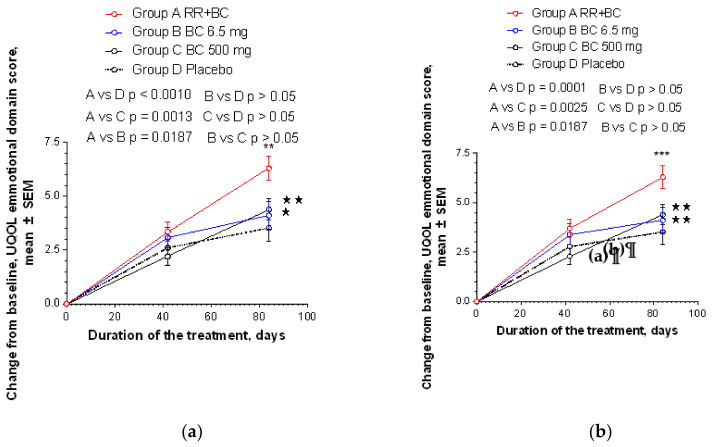
The change from baseline of menopause UQOL emotional domain scores (mean ± SD) over time: (**a**) -ITT analysis, (**b**) -PP analysis. Significance of difference between groups at various time points, calculated by Bonferroni post-test, is expressed by ** *p* < 0.01, *** *p* < 0.001; ns: not significant (A, B, C vs. placebo) and * *p* < 0.05 (B vs. A), ** *p* < 0.001 (B and C vs. A).

## Supplementary Materials

The following are available online at https://www.mdpi.com/1424-8247/13/5/102/s1, Supplementary material 1 (Figures S1–S4: Tables S1–S7) and Supplementary material 2. The datasets used in the current study are available in Supplementary material 2 (Datasheets 1–7: Demographic, Screening, MRS, KMI, UQOL, Adverse Events and Dropouts, Compliance).

## Abbreviations

AE	Adverse event
BC	Black cohosh capsules
BC500	Black cohosh capsules, 500 mg
CRF	Case report form
EC	Ethics committee
GCP	Good Clinical Practice
ICD	International Classification of Diseases
KMI	Kupperman Indexes
MRS	Menopause Relief Score
RR-BC	Rhodiola-Black cohosh combination (Menopause Relief EP^®^) capsules
UQOL	Utian Quality of Life Scale

## Appendix A. ITT and PP Statistics.

**Table A1:** The Menopause Syndrome-related Quality of Life MRS, KI, and UQOL rating scales, and climacteric symptoms used for assessment of efficacy of BC, BC500, and RR-BC on quality of life across the full range of severity of complaints in aging women.

Score/Subscale	Menopause UQOL Scale Item Nr	KI Scale Item Nr	MRS Scale Item Nr	Symptoms Group	Climacteric Symptoms
Vasomotor		1,2 3	1 3	hot flushes, sweating sleep disorders	Sensation of rising heat, outbreaks of sweating Difficulty in falling asleep, difficulty in remaining asleep through the night, waking too early
Psychological/vegetative	12 11, 20 1, 7–10, 16, 21,22	4 5	5 4 6	nervousness/irritability, depressive mood, impaired performance/memory	Nervousness, inner tension, aggressivity Despondency, sadness, tearfulness, lack of drive, mood fluctuations Susceptibility to physical and mental exhaustion, poor concentration, forgetfulness
Somatic		10	2 10	cardiac symptoms joint and muscle symptoms	Palpitations, racing heartbeat, irregular beats, tightness in chest Pain predominantly affecting the finger joints, rheumatic symptoms, itching
Urogenital/Atrophy	15		7 8 9	disorders of sexuality dyspareunia, vaginal dryness	Reduced libido, sexual activity, and satisfaction Symptoms during urination, frequent need to pass urine, accidental incontinence Feeling of dryness of the vagina, symptoms during sexual intercourse
		6		vertigo	sensation of whirling and loss of balance
		7		asthenia	weakness
		9		headache	
	12			anxiety	

**Table A2:** (**a**). The change from baseline of KMI (mean ± SD) over time. All patients including dropouts—ITT analysis. (**b**). The change from baseline of KMI (mean ± SD) over time. Patients completed the treatment and all tests, dropouts excluded—PP analysis.

**(a)**										
**Time after Treatment**	**A, *n* = 55**	**B, *n* = 55**	**C, *n* = 55**	**D, *n* = 55**	**Difference and *p*-Value**					
					**A vs. PL**	**B vs. PL**	**C vs. PL**	**A vs. B**	**A vs. C**	**B vs. C**
Day 0	0	0	0	0	0	0	0	0	0	0
Day 42	12.20 ± 8.99 *n* = 54	9.40 ± 7.28 *n* = 53	10.65 ± 7.14 *n* = 54	5.64 ± 7.77 *n* = 53	6.16 <0.001 ***	3.36 >0.05 ^ns^	4.610 <0.01 **	2.80 >0.05 ^ns^	1.55 >0.05 ^ns^	1.25 >0.05 ^ns^
Day 84	22.35 ± 8.97 *n* = 48	15.58 ± 9.81 *n* = 48	18.42 ± 9.30 *n* = 52	8.80 ± 12.07 *n* = 50	12.55 <0.001 ***	6.78 <0.001 ***	9.62 <0.001 ***	6.77 <0.001 ***	3.93 <0.05 *	2.84 >0.05 ^ns^
Two-way ANOVA					<0.0001 ***	0.0001 ***	<0.0001 ***	<0.0001 ***	0.0203 *	0.0757 ^ns^
**(b)**										
**Time after Treatment**	**A, *n* = 48**	**B, *n* = 48**	**C, *n* = 52**	**D, *n* = 50**	**Difference and *p*-Value**					
					**A vs. PL**	**B vs. PL**	**C vs. PL**	**A vs. B**	**A vs. C**	**B vs. C**
Day 0	0	0	0	0	0	0	0	0	0	0
Day 42	13.50 ± 8.58	10.50 ± 6.70	10.81 ± 7.11	6.08 ± 7.77	7.42 <0.001 ***	4.42 <0.05 *	4.73 <0.01 **	3.00 >0.05 ^ns^	2.69 >0.05 ^ns^	0.31 >0.05 ^ns^
Day 84	22.35 ± 8.97	15.58 ± 9.81	18.42 ± 9.30	8.80 ± 12.07	14.06 <0.001 ***	7.29 <0.001 ***	10.13 <0.001 ***	6.77 <0.001 ***	3.93 <0.05 *	2.84 >0.05 ^ns^
Two-way ANOVA					<0.0001 ***	<0.0001 ***	<0.0001 ***	0.0001 ***	0.0064 **	0.183 ^ns^

Significance of difference between groups at various time points, calculated by Bonferroni post-test, is expressed by * *p* < 0.05, ** *p* < 0.01, *** *p* < 0.001; ns: not significant.

**Table A3:** (**a**). The change from baseline of MRS (mean ± SD) over time. All patients including dropouts—ITT analysis. (**b**). The change from baseline of MRS (mean ± SD) over time. Patients completed the treatment and all tests, dropouts excluded—PP analysis.

**(a)**										
**Time after Treatment**	**A, *n* = 55**	**B, *n* = 55**	**C, *n* = 55**	**D, *n* = 55**	**Difference and *p*-Value**					
					**A vs. PL**	**B vs. PL**	**C vs. PL**	**A vs. B**	**A vs. C**	**B vs. C**
Day 0	0	0	0	0	0	0	0	0	0	0
Day 42	7.18 ± 5.78 *n* = 54	6.17 ± 5.25 *n* = 53	7.44 ± 5.92 *n* = 54	3.81 ± 5.47 *n* = 53	3.37 <0.05 **	2.37 >0.05 ^ns^	3.63 <0.01 **	1.01 >0.05 ^ns^	0.26 >0.05 ^ns^	−1.27 >0.05
Day 84	12.27 ± 6.03 *n* = 48	10.02 ± 7.38 *n* = 48	12.60 ± 7.35 *n* = 52	5.56 ± 7.60 *n* = 50	6.71 <0.001 ***	4.46 <0.001 ***	7.04 <0.001 ***	2.25 >0.05 ^ns^	0.33 >0.05 ^ns^	−2.58 >0.05 ^ns^
Two-way ANOVA					<0.0001 ***	0.0001 ***	<0.0001 ***	>0.05 ^ns^	0.7316 ^ns^	0.0306 *
**(b)**										
**Time after Treatment**	**A, *n* = 48**	**B, *n* = 48**	**C, *n* = 52**	**D, *n* = 50**	**Difference and *p*-Value**					
					**A vs. PL**	**B vs. PL**	**C vs. PL**	**A vs. B**	**A vs. C**	**B vs. C**
Day 0	0	0	0	0	0	0	0	0	0	0
Day 42	8.02 ± 5.57	6.85 ± 5.04	7.65 ± 5.92	3.98 ± 5.56	4.04 <0.001 ***	2.87 <0.05 *	3.67 <0.01 **	1.17 >0.05 ^ns^	0.37 >0.05	−0.8 >0.05 ^ns^
Day 84	12.27 ± 6.03	10.02 ± 7.38	12.60 ± 7.35	5.56 ± 7.68	6.71 <0.001 ***	4.46 <0.001 ***	7.04 <0.001 ***	2.25 >0.05 ^ns^	0.33 >0.05 ^ns^	−2.58 <0.05 *
Two-way ANOVA					<0.0001 ***	0.0001 ***	<0.0001 ***	0.0389 *	0.9808 ^ns^	0.0561 ^ns^

Significance of difference between groups at various time points, calculated by Bonferroni post-test, is expressed by * *p* < 0.05, ** *p* < 0.01, *** *p* < 0.001; ns: not significant.

**Table A4:** (**a**). The change from baseline of UQOL score (mean ± SD) over time. All patients including dropouts—ITT analysis. (**b**). The change from baseline of UQOL score (mean ± SD) over time. Patients completed the treatment and all tests, dropouts excluded—PP analysis.

**(a)**										
**Time after Treatment**	**A, *n* = 55**	**B, *n* = 55**	**C, *n* = 55**	**D, *n* = 55**	**Difference and *p*-Value**					
					**A vs. PL**	**B vs. PL**	**C vs. PL**	**A vs. B**	**A vs. C**	**B vs. C**
Day 0	0	0	0	0	0	0	0	0	0	0
Day 42	9.30 ± 7.92 *n* = 54	7.74 ± 9.56 *n* = 53	7.00 ± 7.51 *n* = 54	5.47 ± 8.28 *n* = 53	3.824 <0.05 *	5.472 >0.05 ^ns^	1.528 >0.05 ^ns^	1.560 <0.05 ^ns^	2.296 >0.05 ^ns^	0.7360 >0.05 ^ns^
Day 84	17.31 ± 10.03 *n* = 48	11.71 ± 11.76 *n* = 48	12.63 ± 8.98 *n* = 52	8.80 ± 10.33 *n* = 50	8.51 <0.001 ***	8.800 >0.05 ^ns^	3.830 >0.05 ^ns^	5.600 <0.01 **	4.680 <0.001 **	0.9200 >0.05 ^ns^
Two-way ANOVA					<0.0001 ***	0.0595 ^ns^	0.0260 *	0.0083 **	0.0032 **	0.943 ^ns^
**(b)**										
**Time after Treatment**	**A, *n* = 48**	**B, *n* = 48**	**C, *n* = 52**	**D, *n* = 50**	**Difference and *p*-Value**					
					**A vs. PL**	**B vs. PL**	**C vs. PL**	**A vs. B**	**A vs. C**	**B vs. C**
Day 0	0	0	0	0	0	0	0	0	0	0
Day 42	10.27 ± 7.78	8.54 ± 9.70	7.27 ± 7.52	5.96 ± 8.216	4.31 <0.05 *	2.58 >0.05 ^ns^	1.31 >0.05 ^ns^	1.73 >0.05 ^ns^	3.00 >0.05 ^ns^	1.27 >0.05 ^ns^
Day 84	17.31 ± 10.03	11.71 ± 11.76	12.63 ± 8.98	8.80 ± 10.33	8.51 <0.001 ***	2.91 >0.05 ^ns^	3.83 <0.05 *	5.6 <0.01 **	4.68 <0.01 **	−0.92 >0.05 ^ns^
Two-way ANOVA					<0.0001 ***	0.0573 ^ns^	0.0382 *	0.0110 *	0.0018 *	0.897 ^ns^

Significance of difference between groups at various time points, calculated by Bonferroni post-test, is expressed by * *p* < 0.05, ** *p* < 0.01, *** *p* < 0.001; ns: not significant.

**Table A5:** (**a**). The change from baseline of UQOL physical health domain score (mean ± SD) over time. All patients including dropouts—ITT analysis. (**b**). The change from baseline of UQOL physical health domain score (mean ± SD) over time. Patients completed the treatment and all tests, dropouts excluded.

**(a)**										
**Time after Treatment**	**A, *n* = 55**	**B, *n* = 55**	**C, *n* = 55**	**D, *n* = 55**	**Difference and *p*-Value**					
					**A vs. PL**	**B vs. PL**	**C vs. PL**	**A vs. B**	**A vs. C**	**B vs. C**
Day 0	0	0	0	0	0	0	0	0	0	0
Day 42	2.87 ± 2.75 *n* = 54	2.64 ± 2.916 *n* = 53	2.29 ± 2.88 *n* = 54	1.26 ± 2.90 *n* = 50	3.83 >0.05 ^ns^	1.378 <0.05 *	1.032 >0.05 ^ns^	0.23 <0.05 ^ns^	0.57 >0.05 ^ns^	0.346 >0.05 ^ns^
Day 84	5.69 ± 3.55	4.23 ± 3.33	3.54 ± 3.90	2.76 ± 3.94	5.36 <0.001 ***	1.689 <0.01 **	0.998 >0.05 ^ns^	1.46 <0.05 *	2.15 <0.001 ***	−0.691 >0.05 ^ns^
Two-way ANOVA					<0.0001 ***	0.0006 ***	0.0278 *	0.0502 ^ns^	0.0026 *	0.247 ^ns^
**(b)**										
**Time after Treatment**	**A, *n* = 48**	**B, *n* = 48**	**C, *n* = 52**	**D, *n* = 50**	**Difference and *p*-Value**					
					**A vs. PL**	**B vs. PL**	**C vs. PL**	**A vs. B**	**A vs. C**	**B vs. C**
Day 0	0	0	0	0	0	0	0	0	0	0
Day 42	3.19 ± 2.75	2.92 ± 2.93	2.38 ± 2.90	1.60 ± 2.74	1.59 <0.05 *	1.32 <0.05 *	0.785 >0.05 ^ns^	0.271 >0.05 ^ns^	0.80 >0.05 ^ns^	0.53 >0.05 ^ns^
Day 84	5.69 ± 3.55	4.23 ± 3.33	3.54 ± 3.90	2.76 ± 3.94	2.928 <0.001 ***	1.47 <0.05 *	7.778 >0.05 ^ns^	1.459 <0.05 *	2.15 <0.05 ***	0.69 >0.05 ^ns^
Two-way ANOVA					<0.0001 ***	0.0031 **	0.1037 ^ns^	0.0587 ^ns^	0.0018 **	0.192 ^ns^

Significance of difference between groups at various time points, calculated by Bonferroni post-test, is expressed by * *p* < 0.05, ** *p* < 0.01, *** *p* < 0.001; ns: not significant.

**Table A6:** (**a**). The change from baseline of UQOL emotional health domain score (mean ± SD) over time. All patients including dropouts—ITT analysis. (**b**). The change from baseline of UQOL emotional health domain score (mean ± SD) over time. Patients completed the treatment and all tests, dropouts excluded—PP analysis.

**(a)**										
**Time after Treatment**	**A, *n* = 55**	**B, *n* = 55**	**C, *n* = 55**	**D, *n* = 55**	**Difference and *p*-Value**					
					**A vs. PL**	**B vs. PL**	**C vs. PL**	**A vs. B**	**A vs. C**	**B vs. C**
Day 0	0	0	0	0	0	0	0	0	0	0
Day 42	3.35 ± 3.27 *n* = 54	3.07 ± 3.58 *n* = 53	2.20 ± 3.04 *n* = 54	2.60 ± 3.79 *n* = 53	0.75 >0.05 ^ns^	0.47 >0.05 ^ns^	−0.4 >0.05 ^ns^	0.28 <0.05 ^ns^	1.15 >0.05 ^ns^	0.87 >0.05
Day 84	6.29 ± 3.94 *n* = 48	4.10 ± 4.44 *n* = 48	4.38 ± 3.58 *n* = 52	3.52 ± 4.46 *n* = 50	2.772 <0.01 ***	0.584 >0.05 ^ns^	0.865 >0.05 ^ns^	0.277 <0.01 **	1.91 <0.01 **	−0.281 >0.05 ^ns^
Two-way ANOVA					<0.001 **	0.3440 ^ns^	0.648	0.0187 *	0.001 **	0.56 ^ns^
**(b)**										
**Time after Treatment**	**A, *n* = 48**	**B, *n* = 48**	**C, *n* = 52**	**D, *n* = 50**	**Difference and *p*-Value**					
					**A vs. PL**	**B vs. PL**	**C vs. PL**	**A vs. B**	**A vs. C**	**B vs. C**
Day 0	0	0	0	0	0	0	0	0	0	0
Day 42	3.71 ± 3.27	3.40 ± 3.62	2.29 ± 3.06	2.78 ± 3.83	0.93 >0.05 ^ns^	0.62 >0.05 ^ns^	–0.49 >0.05 ^ns^	3.31 >0.05 ^ns^	1.148 >0.05 ^ns^	1.11 >0.05 ^ns^
Day 84	6.29 ± 3.94	4.10 ± 4.44	4.38 ± 3.58	3.52 ± 4.46	2.772 <0.001 ***	0.584 >0.05 ^ns^	0.865 >0.05 ^ns^	2.188 <0.01 **	1.91 <0.01 **	–0.28 >0.05 ^ns^
Two-way ANOVA					0.0001 ***	0.3076 ^ns^	0.7236 ^ns^	0.0250 *	0.0013 **	0.430 ^ns^

Significance of difference between groups at various time points, calculated by Bonferroni post-test, is expressed by * *p* < 0.05, ** *p* < 0.01, *** *p* < 0.001; ns: not significant.

**Table A7:** (**a**). The change from baseline of UQOL sexual activity score (mean ± SD) over time. All patients including dropouts—ITT analysis. (**b**). The change from baseline of UQOL sexual activity score (mean ± SD) over time. Patients completed the treatment and all tests, dropouts excluded—PP analysis.

**(a)**										
**Time after Treatment**	**A, *n* = 55**	**B, *n* = 55**	**C, *n* = 55**	**D, *n* = 55**	**Difference and *p*-Value**					
					**A vs. PL**	**B vs. PL**	**C vs. PL**	**A vs. B**	**A vs. C**	**B vs. C**
Day 0	0	0	0	0	0	0	0	0	0	0
Day 42	1.074 ± 2.0 *n* = 54	1.075 ± 2.53 *n* = 53	0.518 ± 2.27 *n* = 54	0.72 ± 1.76 *n* = 53	0.202 >0.05 ^ns^	0.355 >0.05 ^ns^	−0.20 >0.05 ^ns^	0.001 >0.05 ^ns^	0.556 >0.05 ^ns^	0.557 >0.05 ^ns^
Day 84	1.96 ± 2.74 *n* = 48	1.75 ± 3.19 *n* = 48	1.50 ± 2.45 *n* = 52	1.00 ± 2.08 *n* = 50	0.50 >0.05 ^ns^	0.75 >0.05 ^ns^	0.50 >0.05 ^ns^	0.21 >0.05 ^ns^	0.46 >0.05 ^ns^	−0.25 >0.05 ^ns^
Two-way ANOVA					>0.05 ^ns^	>0.05 ^ns^	>0.05 ^ns^	>0.05 ^ns^	>0.05 ^ns^	>0.05 ^ns^
**(b)**										
**Time after Treatment**	**A, *n* = 48**	**B, *n* = 48**	**C, *n* = 52**	**D, *n* = 50**	**Difference and *p*-Value**					
					**A vs. PL**	**B vs. PL**	**C vs. PL**	**A vs. B**	**A vs. C**	**B vs. C**
Day 0	0	0	0	0	0	0	0	0	0	0
Day 42	1.21 ± 2.03	1.19 ± 2.63	0.54 ± 2.31	0.78 ± 1.77	0.43 >0.05 ^ns^	0.41 >0.05 ^ns^	−0.24 >0.05 ^ns^	0.02 >0.05 ^ns^	0.67 >0.05 ^ns^	0.65 >0.05
Day 84	1.96 ± 2.74	1.75 ± 3.19	1.50 ± 2.45	1.00 ± 2.09	0.96 <0.05 *	0.75 >0.05 ^ns^	0.50 >0.05 ^ns^	−0.21 >0.05 ^ns^	0.46 >0.05 ^ns^	0.25 >0.05
Two-way ANOVA					0.027	0.101 ^ns^	0.669 ^ns^	0.766 ^ns^	0.097 ^ns^	0.23 ^ns^

Significance of difference between groups at various time points, calculated by Bonferroni post-test, is expressed by * *p* < 0.05, ** *p* < 0.01, *** *p* < 0.001; ns: not significant.
