# Supplementary material for: Actaea racemosa L. Is More Effective in Combination with Rhodiola rosea L. for Relief of Menopausal Symptoms: A Randomized, Double-Blind, Placebo-Controlled Study
Source: Pharmaceuticals (Basel). 2020 May 21;13(5):102. doi: 10.3390/ph13050102 (PMC7281162; doi:10.3390/ph13050102)
Supplement: Supplementary file 1 [file pharmaceuticals-13-00102-s001.zip › supple - update/pharmaceuticals-790729 supple 1 .pdf]

Supplement 1

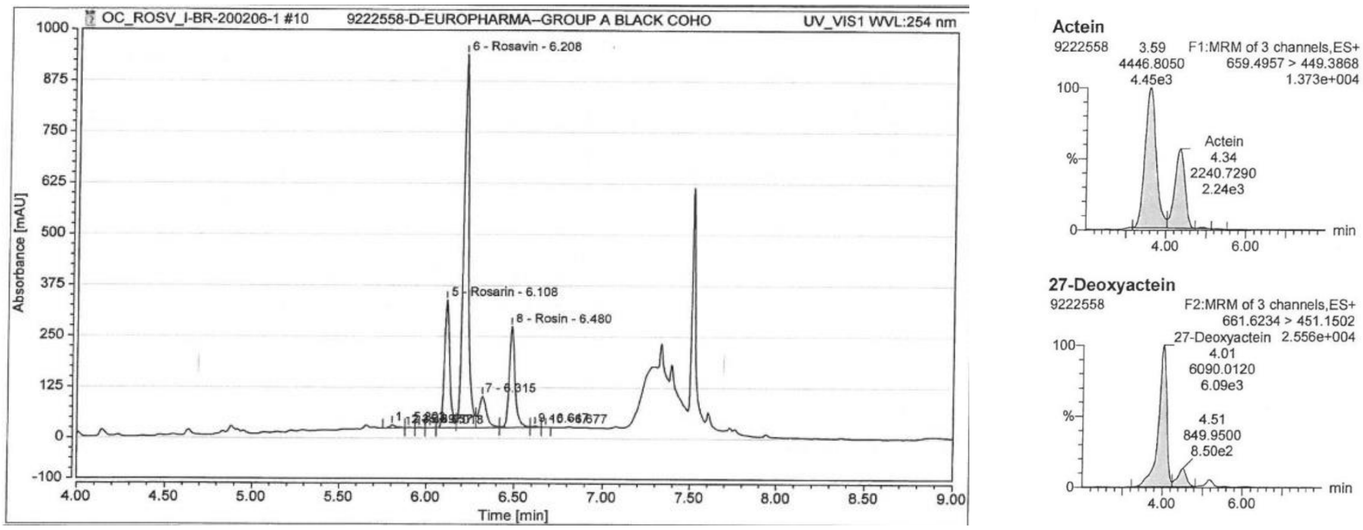

Fig. S1. HPLC fingerprints of Menopause relief EP capsules (batch # 180366, analytical sample 9222558), showing the presence of A – phenylpropanoids rosavin (6.2 min) rosarin (6.1 min) and rosin (6.5 min) from *Rodiola rosea* EP-7 extract (200 mg) detected at 254 nm, and B - triterpene glycosides 27-deoxyactein (4.01 min) and actein (4.34 min) from *Cimicifuga racemose* EP-40 extract (6.5 mg) detected with Waters Xevo TQ MS-WRB 13090 tandem quadrupole mass-spectral detector. Results, mg per serving size (2 capsules): rosavin -12.2, rosarin – 4.6, rosin - 2.6, actein - 0.0135, 27-deoxyactein – 0.0067.

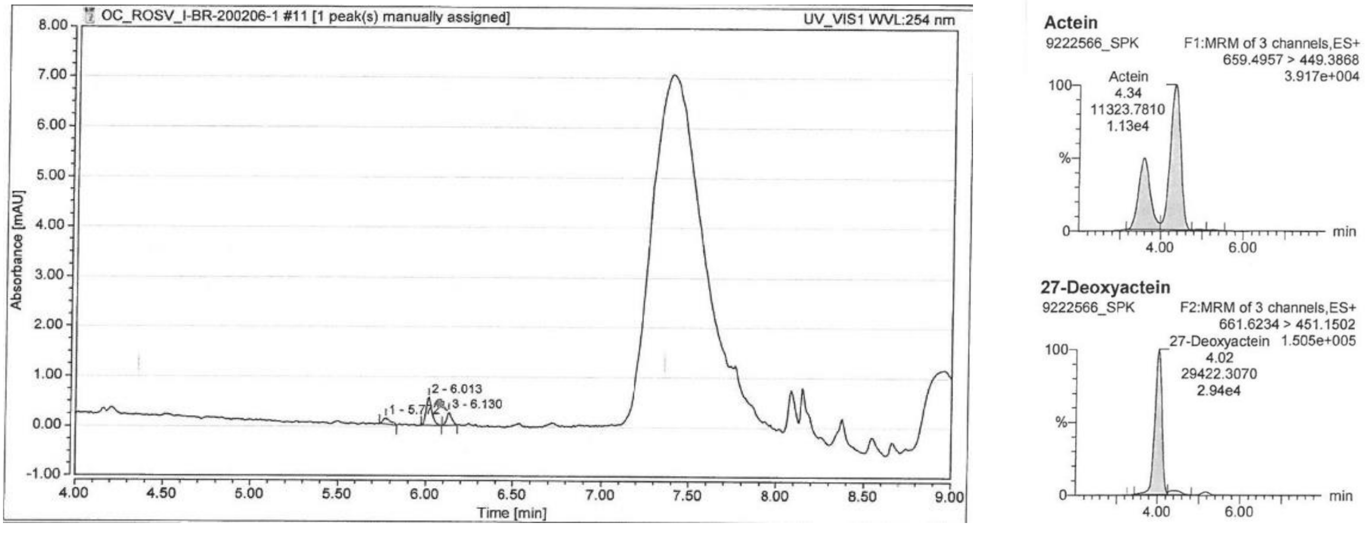

Fig. S2. HPLC fingerprints of *Cimicifuga racemose* EP-40 capsules, 6.5 mg (batch # 180367, analytical sample 9222556), showing A – the lack of phenylpropanoids and B – the presence of triterpene glycosides 27-deoxyactein (4.02 min) and actein (4.34 min) from *Cimicifuga racemose* EP-40 extract. Results, mg per serving size (2 capsules): actein - 0.0169, 27-deoxyactein – 0.0110.

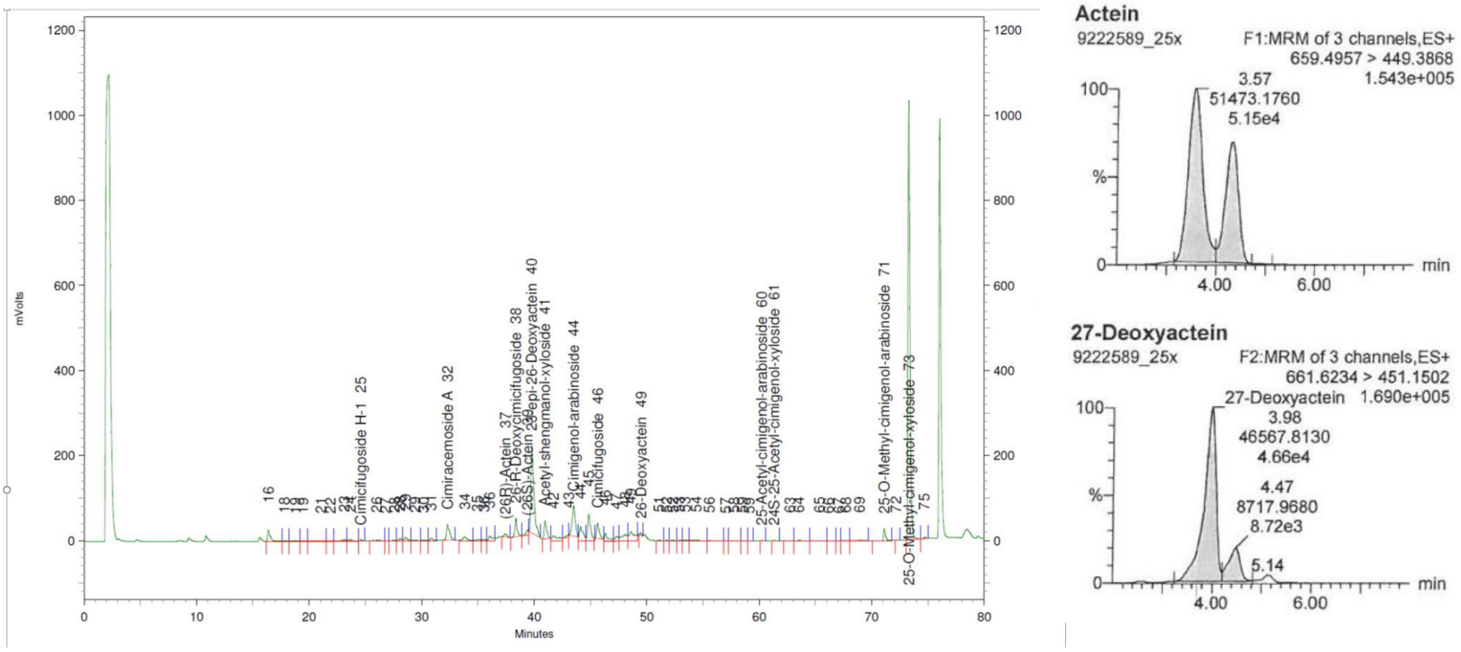

Fig. S3. A- HPLC profile of *Cimicifuga racemose* EP-40 capsules, 500 mg (batch # 180368) detected with LC Shimadzu 4 ELSD 2018 evaporative light scattering detector, acquired and processed with EZChem Elite Version 3.3.3 SP2; the results of analysis are shown in the table 1 below. **B** - triterpene glycosides 27-deoxyactein (4.02 min) and actein (4.34 min) detected with Waters Xevo TQ MS-WRB 13090 tandem quadrupole mass-spectral detector, reverse phase HSS T3 UPLC column and formic acid as the mobile phase. Results, mg per serving size (2 capsules): actein - 1.91, 27-deoxyactein – 2.19.

Table S1. Results of analysis of *Cimicifuga racemose* EP-40 capsules, 500 mg (batch # 180368,

Results

Pk #	Time	Name	ESTD conc	Area	Area %
25	24.612	Cimicifugoside H-1	20.577	29414	0.10
32	32.291	Cimiracemoside A	123.305	696334	2.37
37	37.434	(26R)-Actein	50.225	142385	0.48
38	38.369	26-R-Deoxycimicifugoside	112.333	590595	2.01
40	39.397	(26S)-Actein	61.892	205960	0.70
41	39.811	23-epi-26-Deoxyactein	328.718	3939255	13.42
44	40.962	Acetyl-shengmanol-xyloside	119.390	657732	2.24
46	43.521	Cimigenol-arabinoside	180.247	1362145	4.64
49	45.639	Cimicifugoside	112.475	591917	2.02
60	49.480	26-Deoxyactein	34.544	73481	0.25
61	60.244	25-Acetyl-cimigenol-arabinoside	9.134	7001	0.02
71	61.301	24S-25-Acetyl-cimigenol-xyloside	16.039	18937	0.06
73	71.105	25-O-Methyl-cimigenol-arabinoside	81.680	336288	1.15
	73.310	25-O-Methyl-cimigenol-xyloside	654.005	13286545	45.25

Adverse Events (AEs)

Brief summary of adverse events

In general, during the study period (84 ±6 days) the treatment was well tolerated and only few cases of non-serious adverse events have been reported in study subjects. These AEs were the following: stomach ache-12 cases; increasing of hot flashes or worsening of existing ones-5 cases; increasing of sweating -2 cases; allergic reactions (itching) -2 cases; vomiting -2 cases; nausea -2 cases; head ache -1 case; increasing of appetite-1 case; anxiety-1 case; sleep disturbances-1 case; increasing of heart beating-tachicardia-1 case; hypertension-1 case; diarrhea-1 case.

In total, these AEs were reported in 21 subjects out of 220 participants (9.5%). Only one adverse event was experienced by 14 participants and combinations of them were reported in 7 participants. The types of adverse events were the same in all groups.

A serious adverse events were not observed.

Display of adverse events

Basically, the groups were identical regarding to average age, BMI, duration of climacteric syndrome, concomitant disease and treatment (some subjects were taking levothyroxine, metformin, captopril, amlodipine, vitamin D due to hyperthyrosis, hyperinsulinemia, hypertension and vitamin D deficiency accordingly before enrollment and during the study period). 5 types of AEs were revealed in A Group, 4 types- in B Group, 5 types –in C Group. Number of AEs was higher in Placebo group -8 types, but not statistically significant difference was detected between investigation products (Groups A, B and C) and placebo (Group D) with regard to type, frequency and severity of AEs.

Due to adverse events 2 subjects were dropped out from study in A group, 4 subjects-from B group, 1 subject -from C group and 3subjects-from D group.

Analysis of adverse events

Analysis of adverse events observed in all treatment and Placebo groups shows that:

- During study period the subjects experienced only few adverse events ;
- The adverse events were not serious and not needed treatment;
- The types adverse events are the same in all groups;
- Most adverse events are reported in Placebo group;
- There is not statistically significant difference with regard to type, frequency and severity of adverse events between groups.
- The all above mentioned indicates that none of adverse events are related to the investigational product.

It can be concluded that Adverse events identified during study period are not related to the treatment.

Deaths, Other Serious Adverse Events, and Other Significant Adverse Events

Deaths, other serious adverse events, and other significant adverse deserve special attention have not been observed in this study.

Safety Conclusions

The treatment was well tolerated. Serious adverse events were not observed. Safety of investigational product is high.

Table S2. Adverse events: treatment emergent signs and symptoms (those not seen at baseline and those that worsened even if present at baseline).

Treatment group A , N=55

AEs	Mild		Moderate		Severe		Total		Total
	**Related**	**NR**	**Related**	**NR**	**Related**	**NR**	**Related**	**NR**	**6 (10.9%)**
Head ache			1 (1.8%) N12 (possible)				1(1.8%)		1(1.8%)
Increasing of hot flashes (present at baseline and worsened)			2 (3.6%) N25 (unlikely) 1 (1.8%) N30 (unlikely) 1 (1.8%) N30 (unlikely)				2(3.6%)<br>1(1.8%)		2(3.6%)<br>1(1.8%)
Increasing of sweating (hyperhydrosis)									
Stomach ache	1(1.8%) N37 (possible)		1 (1.8%) N103 (possible)				2(3.6%)		2(3.6%)

Treatment group B, N=55

AEs	Mild		Moderate		Severe		Total		Total
	**Related**	**NR**	**Related**	**NR**	**Related**	**NR**	**Related**	**NR**	**7 (12.7%)**
Stomach ache	3(5.5%) N86 (probable) N96 (probable) N196 (possible)		1(1.8%) N43 (possible)				4(7.3%)		4(7.3%)
Vomiting	1(1.8%) N173 (possible)						1(1.8%)		1(1.8%)
Nausea	1(1.8%) N173 (possible)						1(1.8%)		1(1.8%)
Allergic reaction (itching)	1(1.8%) N173 (possible)						1(1.8%)		1(1.8%)

Treatment group C, N=55

**AEs**	**Mild**		**Moderate**		**Severe**		**Total**		**Total**
	**Drug Related**	**NR**	**Drug Related**	**NR**	**Drug Related**	**NR**	**Drug Related**	**NR**	**7 (12.7%)**
Increase of appetite					1(1.8%) N8 (probable)		1(1.8%)		1(1.8%)
Anxiety			1(3.6%) N11 (probable)				1(1.8%)		1(1.8%)
Increasing of heart beating (tachycardia)	1(1.8%) N36 (possible)						1(1.8%)		1(1.%)
Stomach ache	2(3.6%) N36 (possible) N191 (possible)		1(1.8%) N208 (possible)				3(5.5%)		3(5.5%)
Diarrhea			1(1.8%) N36 (possible)				1(1.8%)		1(1.8%)

Treatment group D, N=55

**AEs**	**Mild**		**Moderate**		**Severe**		**Total**		**Total**
	**Placebo Related**	**NR**	**Placebo Related**	**NR**	**Placebo Related**	**NR**	**Placebo Related**	**NR**	**11 (20%)**
Sleep disturbances	1(1.8%) N45 (probable)						1(1.8%)		1(1.8%)
Increase of hot flashes (present at baseline and worsened)	1(1.8%) N45 (unlikely)		1(1.8%) N26 (unlikely)		1(1.8%) N47 (unlikely)		3(5.5%)		3(5.5%)
Increasing of sweating					1(1.8%) N47 (unlikely)		1(1.8%)		1(1.8%)
Hypertension			1(1.8%) N47 (unlikely)				1(1.8%)		1(1.8%)
Stomach ache	1(1.8%) N130 (possible)		1(1.8%) N42 (possible)				2(3.6%)		2(3.6%)
Vomiting	1(1.8%) N130 (possible)						1(1.8%)	1(1.8%)	1(1.8%)
Nausea	1(1.8%) N130 (possible)						1(1.8%)		1(1.8%)
Allergic reaction (itching)			1(1.8%) N81 (possible)				1(1.8%)		1(1.8%)

Table S3. Adverse events observed in treatment groups

.

Treatment Group	Patients' acronyms	Adverse events	treatment discontinuation due to AE	Total number of patients with AE
**A**	TamKan	Head ache (from starting the treatment, during several days)		5 (9.1%)
**A**	TinTin	Increasing of hot flashes (after 5 days from starting the treatment, during 2 weeks)		
**A**	MziTso	Increasing of hot flashes and sweating (after 2 months from starting the treatment during several days)		
**A**	AnaGav	Stomach ache (after 2 months from starting the treatment during several days)		
**A**	NanKar	Stomach ache (from starting the treatment)	Yes	
**B**	MarTur	Stomach ache (from starting the treatment periodically)	Yes	5 (9.1%)
**B**	TinGum	Stomach ache (after 1 week from starting the treatment)	Yes	
**B**	RusSak	Stomach ache (after 5 days from starting the treatment,)	Yes	
**B**	NatGog	Skin rash /pruritis,<br>vomiting,<br>nausea (after 8 days from starting the treatment)	Yes	
**B**	RusPap	Stomach ache (2 episods) (after 3 days from starting the treatment, during 3 and 5 days)		
**C**	MaiKed	Increase of appetite (after 8 days from starting the treatment, during the whole study)		5 (9.1%)
**C**	KhaKup	Anxiety (from starting the treatment, during 8 days)		
**C**	KhaTke	Increasing of heart beating (from starting the treatment, during 5 days), stomach ache (after 7 weeks from starting the treatment, during 6 days)		
**C**	TamKap	Stomach ache (after 6 weeks from starting the treatment, during 2 weeks)	Yes	
**C**	DarGur	Stomach ache (after 2 days from starting the treatment, during 10 days), diarrhea (after 2 days from starting the treatment, during 2 days)		
**D**	MaiNiz	Increasing of hot flashes (after 3 days from starting the treatment, during 13 days)		6 (10.9)
**D**	KhaLil	Stomach ache (after 1 day from starting the treatment during 3 days)		
**D**	MziBeg	Increasing of hot flashes and sleep disturbances (from starting the treatment, during 28 days)	Yes	
**D**	NinZed	Hypertension, (after 2 days from starting the treatment),<br>Increasing of hot flashes and sweating (after 2 months from starting the treatment)		
**D**	TamSid	Allergic reaction ache (after 2 days from starting the treatment)	Yes	
**D**	NazAvd	Stomachache,<br>vomiting,<br>nausea (after 2 days from starting the treatment, during 5 days).	Yes	

Table S4. Adverse events resulting in treatment discontinuation

Patient ID	Group	AE	Grade	Treatment related	Disease related	Resolved	Narratives
**NanKar**	A	Gastrointestinal pain	**Mild**	possible	No	Partially	Patient had multiple lymphogenic lung lesions with respiratory complaints and pericardial effusion at accrual. The symptoms became more severe after the 3rd treatment cycle. After consulting with a cardiologist the patient was withdrawn from the study.
**MarTur**	B	Gastrointestinal pain					Patient had multiple liver metastases at accrual and slightly elevated transaminases at accrual. After 10 injections of study drugs, the treatment was discontinued due to hepatic failure.
**TinGum**	B	Gastrointestinal pain					The patient was withdrawn. According to investigators the event was considered "intolerance" to taxanes.
**RusSak**	B	Gastrointestinal pain					Patient had an extensive medical history, including hypertension, ischemic coronary disease, and development of pneumonia after a respiratory infection. Withdrawn from the trial.
**NatGog**	B	Skin rush/ pruritus					Patient had chest pain before visit 12 and the ECG was suspicious of myocardial infarction. Patient was referred to a cardiology clinic, where detailed examinations did not reveal infarction, but suggested paclitaxel cardiotoxicity. Patient withdrawn from the trial following cardiologist recommendations.
		Vomiting					
		Nausea					
**TamKap**	C	Gastrointestinal pain					
**MziBeg**	D	hot flashes					
		sleep disturbances					
**TamSid**	D	Skin rush/ pruritus					
**NazAvd**	D	Stomachache					
		vomiting,					
		nausea					

Table S5 Dropout List

Group	Subject's acronym	Code	Start date	Dropout date	Time to treatment failure TTF	Capsules consumed	Expected to consume	Compli-ance, %	Reason
A	TAMKAN	12	30.06.18	10.08.18	42	84	82	102	No clinical improvement
A	MANROD	27	15.09.18	26.10.18	42	98	82	110	No clinical improvement
A	ASKGUM	84	14.11.18	25.12.18	41	20	82	24	No clinical improvement
A	IRAGUM	85	14.11.18	25.12.18	41	60	82	73	No clinical improvement
A	NANKAR	103	08.12.18	01.03.19	82	30	164	18	Adverse event (stomachache)
A	IZEKUC	125	22.01.19	22.03.19	60	114	120	95	No clinical improvement
A	CIAURI	169	14.03.19	25.04.19	42	84	84	100	No clinical improvement
B	MARTUR	43	16.10.18	24.12.18	68	132	136	97	No clinical improvement and adverse event (stomachache)
B	RUSMET	78	06.11.18	26.11.18	20	42	40	105	No clinical improvement
B	TINGUM	86	14.11.18	25.12.18	41	20	82	24	Adverse event (stomachache)
B	RUSSAK	96	27.11.18	08.01.19	41	10	82	12	Adverse event (stomachache)
B	MAIMAC	102	08.12.18	01.03.19	82	10	164	6	Unexplained reason
B	TAMJAN	160	05.03.19	16.04.19	41	70	82	85	No clinical improvement
B	NATGOG	173	19.03.19	04.04.19	45	30	90	33	Adverse event (allergic rush, vomiting, nausea)
C	NELBID	60	24.10.18	04.12.18	40	55	80	69	No clinical improvement
C	LUBCHO	111	15.12.18	18.03.19	84	?	168	-	The patient missed the visits, it was impossible to contact with her
C	TAMKAP	191	24.04.19	24.06.19	60	96	120	80	Adverse event (stomachache)
D	MZIBEG	45	16.10.18	20.11.18	34	48	68	71	Adverse event (Increasing of hot flashes and sleep disturbances)
D	GIUBID	59	24.10.18	04.12.18	40	84	80	105	No clinical improvement
D	NANVAC	72	31.10.18	24.12.18	54	108	108	100	No clinical improvement
D	TAMSID	81	07.11.18	15.11.18	7	6	14	43	Adverse event (allergic reaction)
D	NAZAVD	130	30.01.19	12.02.19	12	12	24	50	Adverse event (stomachache, nausea, vomiting)

Completed = 198 (A-48, B-48, C-52, D-50), Dropouts = 22 (A-7, B-7, C-3, D-5)

Group A – 7 patients (6 - no clinical improvement, 1 – adverse events);
Group B – 7 patients, (2 – no clinical improvement, 3 – adverse events, 1 – no clinical improvement and adverse events, 1 – unexplained reason);
Group C – 3 patients (1 –no clinical improvement, 1 –unexplained reason, 1 –adverse events);
Group D – 5 patients (2 –no clinical improvement, 3 –adverse events).

Table S6. Compliance of dropout patients, % is less than estimated limit – 75%

	**A**	**B**	**C**	**D**
	102.4	97.1	68.8	70.6
	109.8	105.0	-	105.0
	24.4	24.4	80.0	100.0
	73.2	12.2		42.9
	18.3	6.1		50.0
	95.0	85.4		
	100.0	33.3		
**Average**	**74.7**	**51.9**	**74.4**	**73.7**

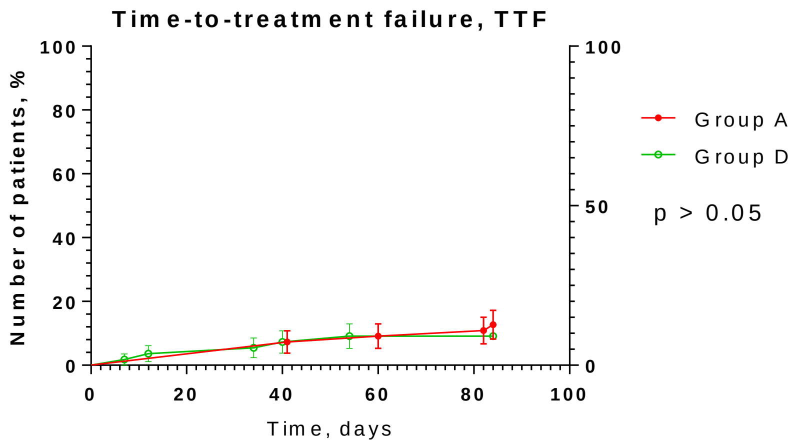

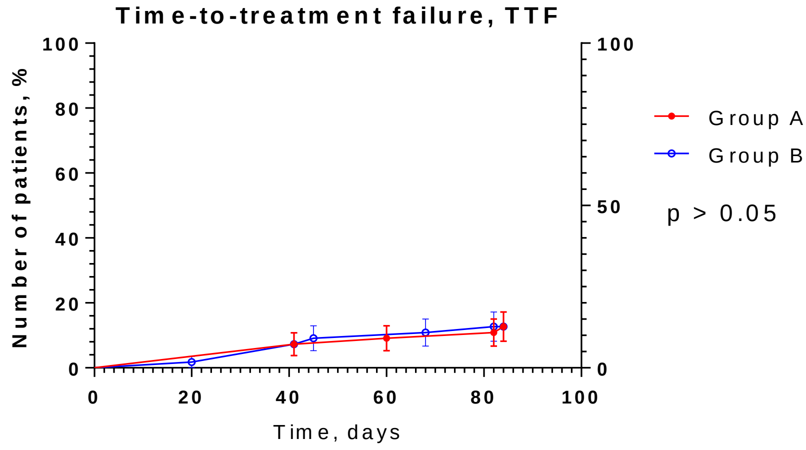

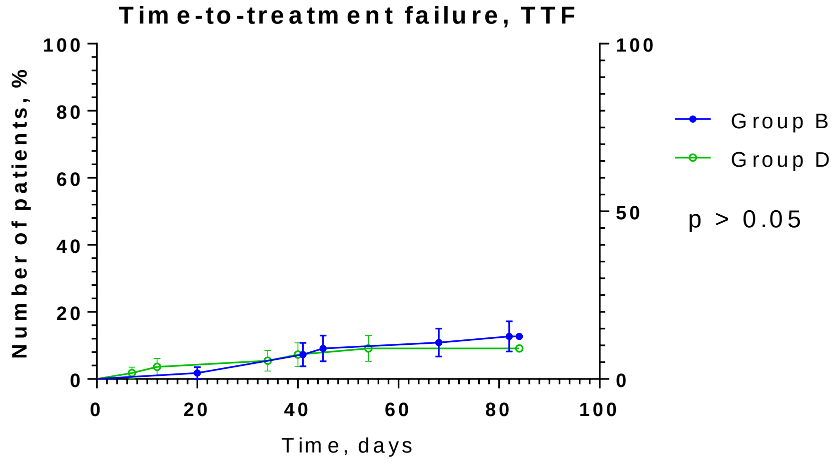

Figure S4 Time to treatment failure

Table S7 CONSORT checklist

Section/Topic	Item No	Checklist item	Reported on page No
**Title and abstract**			
	1a	Identification as a randomised trial in the title	1
	1b	Structured summary of trial design, methods, results, and conclusions (for specific guidance see CONSORT for abstracts)	2
**Introduction**			
Background and objectives	2a	Scientific background and explanation of rationale	4,5
	2b	Specific objectives or hypotheses	6
**Methods**			
Trial design	3a	Description of trial design (such as parallel, factorial) including allocation ratio	9, Appendix A table1
	3b	Important changes to methods after trial commencement (such as eligibility criteria), with reasons	n/a
Participants	4a	Eligibility criteria for participants	9
	4b	Settings and locations where the data were collected	8.9
Interventions	5	The interventions for each group with sufficient details to allow replication, including how and when they were actually administered	9,10, Appendix B
Outcomes	6a	Completely defined pre-specified primary and secondary outcome measures, including how and when they were assessed	12,13, Table1 Appendix C
	6b	Any changes to trial outcomes after the trial commenced, with reasons	n/a
Sample size	7a	How sample size was determined	14
	7b	When applicable, explanation of any interim analyses and stopping guidelines	n/a
Randomisation:			
Sequence generation	8a	Method used to generate the random allocation sequence	10
	8b	Type of randomisation; details of any restriction (such as blocking and block size)	10
Allocation concealment mechanism	9	Mechanism used to implement the random allocation sequence (such as sequentially numbered containers), describing any steps taken to conceal the sequence until interventions were assigned	10

Implementation	10	Who generated the random allocation sequence, who enrolled participants, and who assigned participants to interventions	10
Blinding	11a	If done, who was blinded after assignment to interventions (for example, participants, care providers, those assessing outcomes) and how	10,11
	11b	If relevant, description of the similarity of interventions	10
Statistical methods	12a	Statistical methods used to compare groups for primary and secondary outcomes	13
	12b	Methods for additional analyses, such as subgroup analyses and adjusted analyses	Appendix D
**Results**			
Participant flow (a diagram is strongly recommended)	13a	For each group, the numbers of participants who were randomly assigned, received intended treatment, and were analysed for the primary outcome	3, Figures 1-3, Table 1
	13b	For each group, losses and exclusions after randomisation, together with reasons	3
Recruitment	14a	Dates defining the periods of recruitment and follow-up	8
	14b	Why the trial ended or was stopped	n/a
Baseline data	15	A table showing baseline demographic and clinical characteristics for each group	Table 1
Numbers analysed	16	For each group, number of participants (denominator) included in each analysis and whether the analysis was by original assigned groups	3, Figures 1-3, Table 1
Outcomes and estimation	17a	For each primary and secondary outcome, results for each group, and the estimated effect size and its precision (such as 95% confidence interval)	Figures 1-3, Appendix D
	17b	For binary outcomes, presentation of both absolute and relative effect sizes is recommended	Figures 1-3, Appendix D
Ancillary analyses	18	Results of any other analyses performed, including subgroup analyses and adjusted analyses, distinguishing pre-specified from exploratory	n/a
Harms	19	All-important harms or unintended effects in each group	6
**Discussion**			
Limitations	20	Trial limitations, addressing sources of potential bias, imprecision, and, if relevant, multiplicity of analyses	8,
Generalisability	21	Generalisability (external validity, applicability) of the trial findings	8
Interpretation	22	Interpretation consistent with results, balancing benefits and harms, and considering other relevant evidence	8
**Other information**			
Registration	23	Registration number and name of trial registry	6
Protocol	24	Where the full trial protocol can be accessed, if available	14
Funding	25	Sources of funding and other support (such as supply of drugs), role of funders	14
